# A Splicing Mutation in the Novel Mitochondrial Protein DNAJC11 Causes Motor Neuron Pathology Associated with Cristae Disorganization, and Lymphoid Abnormalities in Mice

**DOI:** 10.1371/journal.pone.0104237

**Published:** 2014-08-11

**Authors:** Fotis Ioakeimidis, Christine Ott, Vera Kozjak-Pavlovic, Foteini Violitzi, Vagelis Rinotas, Eleni Makrinou, Elias Eliopoulos, Costas Fasseas, George Kollias, Eleni Douni

**Affiliations:** 1 Department of Biotechnology, Agricultural University of Athens, Athens, Greece; 2 Division of Immunology, Biomedical Sciences Research Center “Alexander Fleming”, Vari, Greece; 3 Department of Microbiology, Biocenter, University of Würzburg, Würzburg, Germany; 4 Department of Crop Science, Agricultural University of Athens, Athens, Greece; Alexander Fleming Biomedical Sciences Research Center, Greece

## Abstract

Mitochondrial structure and function is emerging as a major contributor to neuromuscular disease, highlighting the need for the complete elucidation of the underlying molecular and pathophysiological mechanisms. Following a forward genetics approach with N-ethyl-N-nitrosourea (ENU)-mediated random mutagenesis, we identified a novel mouse model of autosomal recessive neuromuscular disease caused by a splice-site hypomorphic mutation in a novel gene of unknown function, *DnaJC11*. Recent findings have demonstrated that DNAJC11 protein co-immunoprecipitates with proteins of the mitochondrial contact site (MICOS) complex involved in the formation of mitochondrial cristae and cristae junctions. Homozygous mutant mice developed locomotion defects, muscle weakness, spasticity, limb tremor, leucopenia, thymic and splenic hypoplasia, general wasting and early lethality. Neuropathological analysis showed severe vacuolation of the motor neurons in the spinal cord, originating from dilatations of the endoplasmic reticulum and notably from mitochondria that had lost their proper inner membrane organization. The causal role of the identified mutation in *DnaJC11* was verified in rescue experiments by overexpressing the human ortholog. The full length 63 kDa isoform of human DNAJC11 was shown to localize in the periphery of the mitochondrial outer membrane whereas putative additional isoforms displayed differential submitochondrial localization. Moreover, we showed that DNAJC11 is assembled in a high molecular weight complex, similarly to mitofilin and that downregulation of mitofilin or SAM50 affected the levels of DNAJC11 in HeLa cells. Our findings provide the first mouse mutant for a putative MICOS protein and establish a link between DNAJC11 and neuromuscular diseases.

## Introduction

Mitochondria are highly dynamic organelles that have a central role in a plethora of cellular functions like oxidative phosphorylation (OXPHOS), calcium buffering, apoptosis, metabolism and reactive oxygen species generation among others. OXPHOS is the most essential mitochondrial function resulting in the synthesis of ATP. High energy demanding tissues like the central nervous system (CNS) and muscle are sensitive to mitochondrial dysfunction and this explains why mitochondrial diseases very commonly manifest with neuromuscular symptoms [1]. Moreover, many classic neurological diseases are characterized by changes in mitochondrial function, dynamics and morphology [2], [3], [4]. The term mitochondrial disease is generally ascribed to genetic diseases caused by defects in OXPHOS [5]. Recently, more and more neuromuscular diseases that are not directly linked with OXPHOS deficiency are considered mitochondrial diseases because of involvement of other mitochondrial processes like mitochondrial fusion/fission and mitochondrial protein importation [6]. Even though many mitochondrial diseases are multisystemic, the neuromuscular manifestations are usually the most prominent ones. Mitochondrial diseases remain incurable due to the rarity of these diseases and the lack of animal models [7]. Apart from the diseases caused directly by mitochondrial dysfunction, the central role of mitochondrial function is emerging as a converging point in many neuromuscular/neurodegenerative diseases [2] displaying impairment of mitochondrial function, dynamics and structure. Mitochondrial ultrastructural abnormalities have been observed in patients with Alzheimer's disease, Parkinson's disease and Amyotrophic Lateral Sclerosis (ALS) [8], [9]. Mitochondrial dysfunction is now well documented in ALS [10] although it remains under investigation whether mitochondrial dysfunction is a necessary step in neurodegeneration.

Mitochondria have a characteristic structure as they are surrounded by two membranes: the outer mitochondrial membrane (OM) and the inner mitochondrial membrane (IM) defining the intermembrane space (IMS). The IM is further subdivided into the inner boundary membrane (IBM), which is adjacent to the OM, and the cristae membranes (CMs) that protrude into the matrix space [8], [11]. The structures that connect the IBM to the CMs have been termed cristae junctions (CJs) and have been found to be generally similar between mitochondria of different tissues [11]. Of the approximately 1300 identified mitochondrial proteins only 13 are synthesized inside the mitochondrion [12]. The rest are encoded by nuclear genes and have to be imported from the cytosol through a specialized and highly sophisticated protein machinery of the OM (TOM and SAM complexes), the IM (TIM complexes) and the IMS (MIA complex) depending on the targeting of each protein [12]. Mitochondrial proteins involved in mitochondrial fusion or protein translocation are preferentially located in the IBM and OM, whereas proteins involved in OXPHOS are enriched in the cristae membrane. In contrast to the textbook view of mitochondrial cristae structure, these membrane formations are extremely heterogeneous and dynamic according to the cellular environment as it has been revealed through electron tomography [13].

Recently, significant advances have been made in identifying the proteins involved in the biogenesis or maintenance of CMs and CJs. Three independent groups working in yeast identified a protein complex, essential for normal CM morphology that has been given the names of MICOS, MITOS or MINOS [14], [15], [16]. At the same time a homologous complex in human cells was also identified and was termed as Mitochondrial Intermembrane space Bridging (MIB) complex [17]. We will refer to these complexes collectively as MICOS (MItochondrial COntact Site complex). Central components of this complex have been determined to be mitofilin/Fcj1 and MINOS1/Mio10. Knocking down of these proteins almost completely abolishes CJs and alters CMs organization [18], [19]. Another member of the MICOS complex, essential for CMs normal morphology and present both in yeast and human cells is ChChd3/Aim13 [20]. A large number of MICOS members interactor proteins have been identified in yeast and mammalian mitochondria. These include SAM, TOM and MIA complex members, CHCHD6, DNAJC11, UGO1, OPA1, HSPA9, DISC1 and APOOL [16], [19], [21], [22], [23]. Of these, DNAJC11 has an unknown mitochondrial localization and function.

Using a forward genetics approach with random chemical mutagenesis in the mouse we identified a novel autosomal recessive phenotype with distinct neurological and lymphoid symptoms. Neuropathological characterization showed severe vacuolation in the motor neurons of the spinal cord associated with severely disrupted mitochondria and abnormal cristae structure. This phenotype is caused by a splicing point mutation within the *DnaJC11* gene, which encodes a ubiquitously expressed mitochondrial protein that possibly interacts with the MICOS complex. The aim of this study was to characterize the phenotype displayed by the mutant mice as well as to identify and characterize the causal mutation in order to define the underlying molecular mechanism that results in disease pathogenesis.

## Results

### Generation and Clinical Characterization of *spastic* Mice

**Figure 1:**
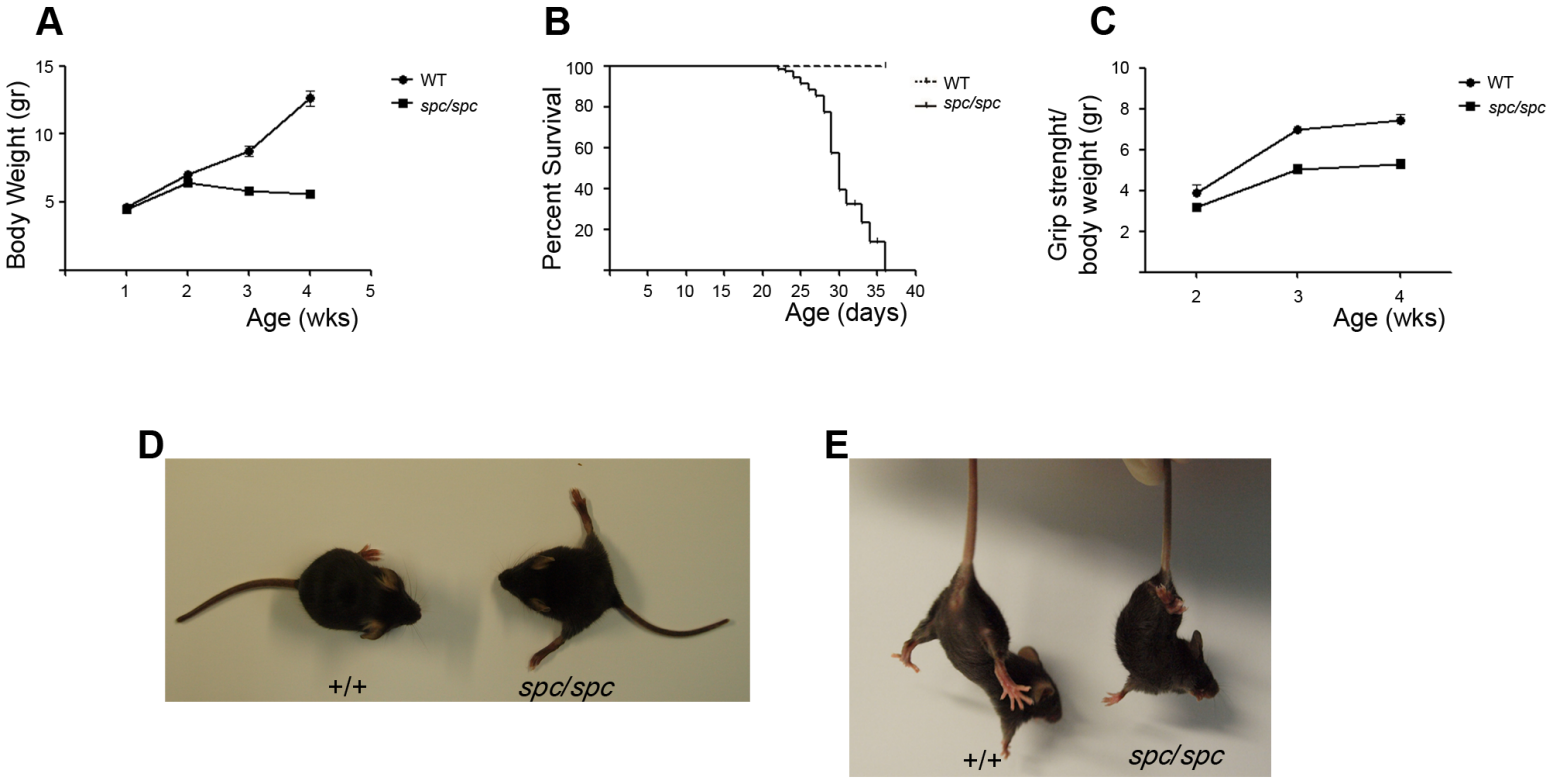
Clinical characterization of the *spastic* phenotype. (A) Body weight curves of spastic (*spc/spc)* mice and sex matched control (+/+ or *spc*/+) littermates (n = 21 per group). (B) Kaplan–Meier survival curve of *spc/spc* mice and control (+/+ or *spc*/+) littermates (n = 38 per group). (C) Grip strength measurements of *spc/spc* mice and control (+/+ or *spc*/+), sex matched, littermates. Grip strength values were normalized with body weight to account for the reduced body size of *spc/spc* mice (n = 16). (D) Abnormal hind limb posture of *spc/spc* mice. (E) Loss of hind limb extension reflex when *spc/spc* mice are suspended by the tail. Mice shown are females and 3 weeks old littermates.

Following chemical mutagenesis with intraperitoneal administration of N-ethyl-N-nitrosourea (ENU) in G0 male mice [24], a novel recessive neuromuscular phenotype was identified in G3 progeny characterized by abnormal hind limb posture, abnormal locomotion, muscle weakness, hind limb clutching when mice are suspended by the tail, spasticity, limb tremor, growth retardation, progressive cachexia and premature death (Figure 1, and Video S1). This is an autosomal recessive mendelian trait with full penetrance that affects both sexes equally. Because of the marked neurological symptoms, this phenotype was designated as *spastic (spc).* Heterozygous mice (*spc/+)* did not differ from their wild-type (WT) littermates.

*Spc*/*spc* mice were indistinguishable from their WT littermates at birth. First symptoms manifested approximately at the tenth day after birth with abnormal hind limb posture and locomotion defect. From the second week, *spc*/*spc* mice stopped gaining weight (Figure 1A) and by the fifth week after birth all the *spc*/*spc* mice had died (Figure 1B). Upon identification of *spc* mice, mashed wet food pellets were added inside the cage, so the observed cachexia and growth retardation was not due to difficulties in accessing food. Death was probably associated with breathing and moving difficulties as well as general wasting. Muscle weakness was progressive and started at the hind limbs continuing with the fore ones, judging by the loss of the limb extension reflex (Figure 1C-E).

### *Spc*/*spc* mice Developed Motor Neuron Pathology in the Spinal Cord

**Figure 2:**
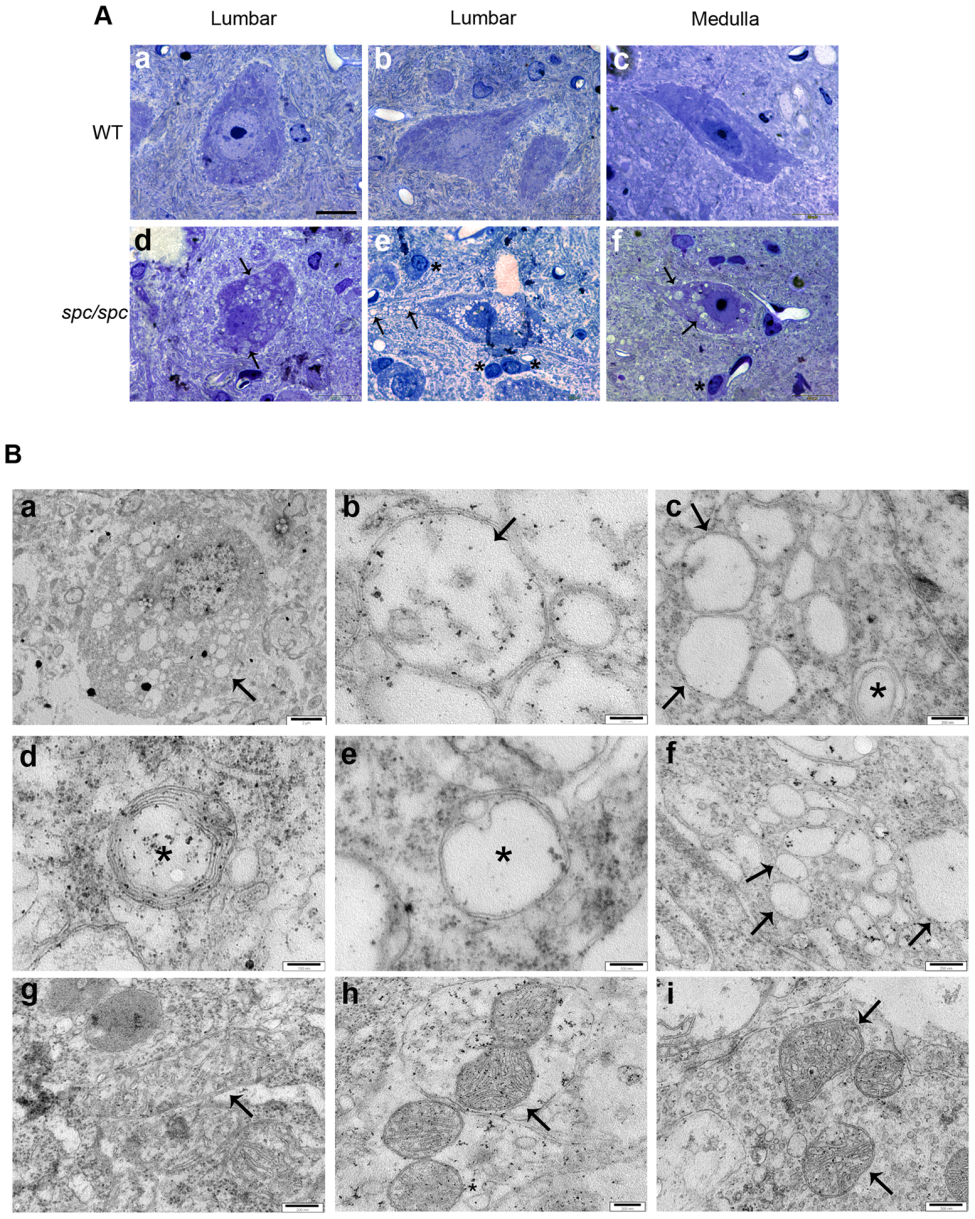
Neuronal vacuolation and abnormal mitochondria in the CNS of *spc/spc* mice. (A) Representative toluidine blue stained resin sections of motor neurons in the lumbar segment of the spinal cord (a,b,d,e) and neurons in the medulla (c,f) from WT littermates (a-c) and *spc/spc* mice (d-f). Lumbar, (n = 5 in three different experiments), medulla (n = 3 in two different experiments). Scalebar, 20 µm. Arrows indicate vacuoles and asterisks glial cells. (B) Representative electron micrographs of motor neuron cell bodies (a-g) and terminal axons (h-i) in the region of the ventral horn of the spinal cord from *spc/spc* (a-f and h) and WT control mice (g and i). (a), low power magnification of a vacuolated motor neuron. (b-g), high power magnification in the cell bodies of motor neurons. (b-e) mitochondria with disrupted cristae (arrows) or others with abnormally stacked or concentric membranes (asterisks) can be identified. Arrows in c indicate double membrane bound vacuoles completely electron transparent, devoid of cristae. (f), single membrane bound vacuoles (arrows) possibly originating from the ER. (g), a normal WT mitochondrion (arrow). (h-i), synaptic mitochondria (arrows) in terminal axons identified by the presence of prosynaptic vesicles. WT (n = 2), *spc/spc* (n = 4). Scalebars: a, 2 µm; b, d-e, 100 nm, c, f-i, 200 nm.

The motor defects observed in *spc/spc* mice prompted us to investigate for motor neuron pathology. Because of the severity of the symptoms in the hind limbs, we firstly examined cross sections of the lumbar segment of the spinal cord from 4 weeks old *spc/spc* mice. Motor neurons in the anterior horn of WT littermates were properly maintained with obvious Nissl substance, nuclei and nucleoli (Figure 2Aa-b), whereas these cells in the *spc*/*spc* mice appeared completely vacuolated (Figure 2Ad-e). These vacuoles were mainly confined in the cell bodies of motor neurons but also extended to dendrites (Figure 2Ae). They were roughly circular and of various sizes and appeared either opaque (Figure 2Ad) or transparent (Figure 2Ae), usually filling the whole body of the neuron. No such vacuoles were observed in the neuropil, the white matter, the dorsal horn neurons or in glial cells of the spinal cord. Nuclei and nucleoli of motor neurons appeared normal (Figure 2A). The same analysis was performed for various regions of the brain. Again, cell structure in the WT mice was well maintained and no vacuolation was observed (Figure 2Ac). In the *spc/spc* mice though, vacuolation was present mainly in the medulla (Figure 2Af), which was affected to a lesser extent compared to the spinal cord with regard to the number of affected neurons and the number of vacuoles in the affected neurons.

To gain insight into the nature of the motor neuron vacuoles, transmission electron microscopy was performed in the ventral horn of lumbar spinal cord. Our results showed that the vacuoles observed in the motor neurons of *spc/spc* mice (Figure 2A) were of dual origin; either mitochondria that had abnormal cristae structure (Figure 2Ba-e) or the endoplasmic reticulum (ER) (Figure 2Bf). In *spc/spc* mice, many mitochondria were at various stages of mitochondrial cristae degeneration (Figure 2Bb), while others were identified as vacuoles bound by double membrane but completely devoid of internal membranes that could represent the most advanced stage of cristae disorganization (Figure 2Bc). Occasionally, abnormal cristae organization resembling concentric cristae were also observed (Figures 2Bd-e and S1). Moreover, all vacuolated mitochondria appeared roughly round, without the characteristic elongated morphology. In completely vacuolated motor neurons, no mitochondria with recognizable internal structure and shape could be observed. In contrast, mitochondria in the bodies of WT control motor neurons had normal appearance and structure, with the characteristic elongated shape and preserved cristae (Figure 2Bg). The abnormal mitochondria were located in the bodies of motor neurons but not in dendrites and synapses (Figure 2Bh). Synaptic mitochondria, identified by the presence of synaptic vesicles, had the same structure as the WT ones (Figure 2Bi). Single membrane bound vacuoles that seemed to originate from dilatations of the cisternae of the ER were also observed frequently (Figure 2Bf).

### Lymphoid and Blood Abnormalities in *spc/spc* Mice

**Figure 3:**
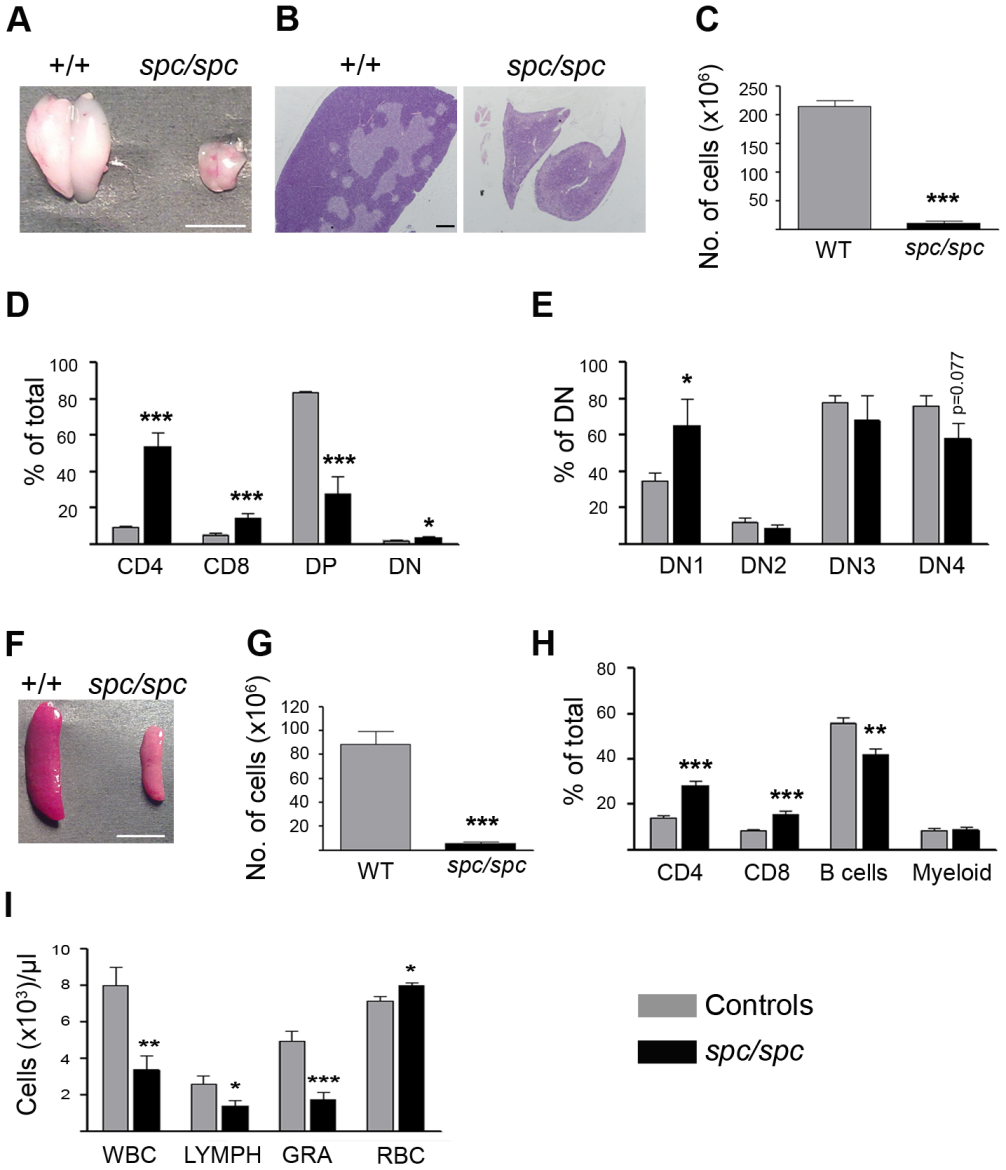
Lymphoid and blood abnormalities in *spc* mice. (A) Representative thymi dissected from *spc/spc* and WT (+/+) littermate mice at 4 weeks of age. Scalebar, 5mm. (B) Representative H/E stained thymic sections from *spc/spc* and +/+ littermate mice (n = 4). Scalebar, 400 µm. (C) Total thymus cellularity in *spc/spc* mice and control littermates, (n = 14 per group). (D) Percentage of thymic subpopulations in *spc/spc* mice and control littermates as determined by flow cytometry after staining of thymocytes with antibodies against CD4 and CD8. Data represent means ± SE from four independent experiments, (n = 13 per group). (E) Percentages of CD4^-^CD8^-^ double negative (DN) subpopulations as determined by flow cytometry after staining of thymocytes with antibodies against CD25 and CD44, in *spc/spc* and control littermates, (n = 6 per group). (F) Representative spleens dissected from *spc/spc* and +/+ littermate mice at 4 weeks of age. Scalebar, 5 mm. (G) Total spleen cellularity of *spc/spc* mice and control littermates, (n = 14 per group). (H) Percentage of splenic subpopulations in *spc/spc* mice and control littermates as determined by flow cytometry using antibodies against CD4, CD8, B220 (B cells), Gr1 and CD11b (Myeloid). Data represent means ± SE from four independent experiments, (n = 10 per group). (I) Peripheral blood counts of *spc/spc* mice and control littermates, (n = 7 per group). Controls presented in bar graphs are healthy littermates (+/+ and *+/spc*).

Apart from the neuromuscular phenotype, *spc/spc* mice developed severe thymic and splenic hypoplasia that started 10 days after birth (Figure 3) showing massive degeneration. At the moribund stage, the thymi of *spc/spc* mice were markedly hypoplastic and had approximately 20% of the WT littermate weight (Figure 3A). Hematoxylin/eosin staining of *spc/spc* thymi revealed loss of organ architecture with great reduction of darkly stained cortical area and an increase of medulla area that had lost its characteristic patchy appearance (Figure 3B). Flow cytometric analysis of thymi revealed not only decreased cellularity (Figure 3C) but also a significant reduction of the percentage of CD4^+^CD8^+^ double positive cells and an approximately four fold increase in the percentage of CD4^+^ and CD8^+^ single positive cells in *spc/spc* mice (Figure 3D). The percentage of CD4^-^CD8^-^ double negative (DN) subpopulation was slightly but statistically significantly increased (3,65% in *spc/spc* compared to 1,98% in controls) in *spc/spc* mice. Analysis of thymi for CD25 and CD44 markers, in order to analyze the DN subpopulations, revealed a significant increase in the percentages of DN1 (CD25^+^CD44^-^) subpopulation in *spc/spc* mice (Figure 3E).

*Spc/spc* mice also developed severe splenic hypoplasia (Figure 3F-G). Flow cytometric analysis of spleens revealed two fold increase in the percentages of CD4^+^ and CD8^+^ single positive cells in *spc/spc* mice (Figure 3H). B220^+^ B cell percentage was reduced by 14% in *spc/spc* mice (42% compared to 56% of WT littermates), whereas myeloid cell percentages, identified as being positive for CD11b, did not differ between *spc/spc* mice and WT littermates (Figure 3H). Absolute cell numbers for all cell populations studied in the spleen and thymus were much lower in the *spc/spc* mice due to the severe hypoplasia (Figure 3C, G).

To better characterize the *spc* phenotype we also performed peripheral blood hematological analysis (Figure 3I and Table 1). Blood counts revealed profound leucopenia in *spc/spc* mice (two-fold decrease), involving both lymphocytes and granulocytes, whereas the number of red blood cells in *spc/spc* mice was found to be slightly increased (Figure 3I). Clinical chemistry analysis (Table 2) showed a four fold increase in enzymes aspartate transaminase (AST) and creatine phosphokinase (CPK) in *spc/spc* mice, indicating possible muscle damage. Lactate dehydrogenase (LDH) was also found statistically significantly elevated in *spc/spc* mice.

**Table 1:** Total Blood Counts in *spc/spc* mice.

Peripheral blood	Controls	*Spc/spc*
WBCs (10^3^/mm^3^)	7.986±1.018	3.357±0.7668 **
Lymphocytes (x10^3^ cells/µl)	2.571±0.4617	1.371±0.2909 *
Lymphocytes (%)	31.29±2.296	41.71±3.053 *
Granulocytes (x10^3^ cells/µl)	4.929±0.5528	1.743±0.4151 ***
Granulocytes (%)	62.57±2.750	51.71±2.868 *
RBCs (10^6^/mm^3^)	7.150±0.2552	7.963±0.1466 *
Hemoglobin (g/dl)	11.64±0.3169	11.66±0.3330
Hematocrit (%)	36.31±1.019	35.19±1.068
RDW (%)	22.24±1.579	21.57±1.399
MCV (fl)	50.93±1.040	44.23±1.338 **
MCH (pg)	16.34±0.2562	14.64±0.3835
MCHC (g/dl)	32.09±0.2824	33.16±0.3484 *
Platelets (10^3^/mm^3^)	475.1±58.07	382.9±44.26
PCT (%)	0.3499±0.04953	0.2661±0.04272
MPV (µm^3^)	7.314±0.3985	6.700±0.4282
PDW (µm^3^)	8.314±1.168	8.400±1.200
MXD	0.4857±0.07377	0.2429±0.08411 p = 0.051
MXD %	6.143±0.8845	6.571±1.192

WBCs: White blood cells, RBCs: Red blood cells, RDW: Red cell distribution width MCV: Mean corpuscular volume, MCH: Mean corpuscular hemoglobin, MCHC: Mean corpuscular hemoglobin concentration, PCT: Plateletcrit, MPV: Mean platelet volume, PDW: Platelet distribution width, MXD: Mixed cell count. Data are expressed as mean ± SE, (n = 7 per group). Control mice (+/+ and *spc*/+) were sex matched littermates. Student's t test was performed for statistical analysis. *p<0.05; **p<0.01; ***p<0.001.

**Table 2:** Clinical chemistry in *spc/spc* mice.

Peripheral blood	Controls	*Spc/spc*
ALP	454.0±104.0	283.5±66.75
ALT	40.80±4.017	117.6±57.17
AST	90.20±10.27	364.0±56.00 **
LDH	391.6±61.81	642.5±56.03 *
CPK (U/L)	208.0±56.32	857.2±139.9 ***
Urea (mg/dl)	122.0±81.39	81.67±23.51
Cholesterol (mg/dL)	98.43±17.52	61.00±6.831
Glucose (mg/dL)	263.0±58.13	241.3±79.90
Total Protein	4.429±0.2055	3.700±0.1366 *
Creatinine (U/L)	0.5840±0.06562	0.6175±0.1006
Lactic Acid (mg/dL)	55.13±6.983	60.85±6.462
Amylase (U/L)	1278±170.9	2529±619.6 p = 0.06
Lipase (U/L)	81.00±21.40	52.33±17.50

ALP: Alkaline Phosphatase, ALT: Alanine Aminotransferase, AST: Aspartate Aminotransferase, LDH: Lactate Dehydrogenase, CPK: Creatine Phosphokinase. Data are expressed as mean ± SE. Male *spc/spc* mice and male control (+/+ and *spc*/+) littermates were used. Amylase (n = 8). LDH, CPK, Urea, Cholesterol, Total Protein and Lactic Acid (n = 7). ALP, Lipase (n = 6). ALT, AST, Creatinine (n = 5). Glucose (n = 3). Student's t test was performed for statistical analysis. *p<0.05; **p<0.01; ***p<0.001.

In order to investigate whether the lymphoid aspect of the *spc* phenotype is involved in the motor deficiencies, *RAG-2* (recombination activating gene) knockout mice were crossed with the *spc/*+ mice to obtain *spc/spc* mice lacking *RAG-2* gene. *RAG-2* gene is necessary for immunoglobulin and T-cell receptor genes rearrangements, so RAG-2 deficient mice lack mature B and T-cells and are immunodeficient [26]. In the double mutant mice, clinical symptoms of the *spc* phenotype were unchanged (n = 5, unpublished data) suggesting that the lymphoid and motor aspects of the *spc* phenotype are two distinct sets of symptoms.

### Identification of the Causal Mutation for the *spc* Phenotype in *DnaJC11*

**Figure 4:**
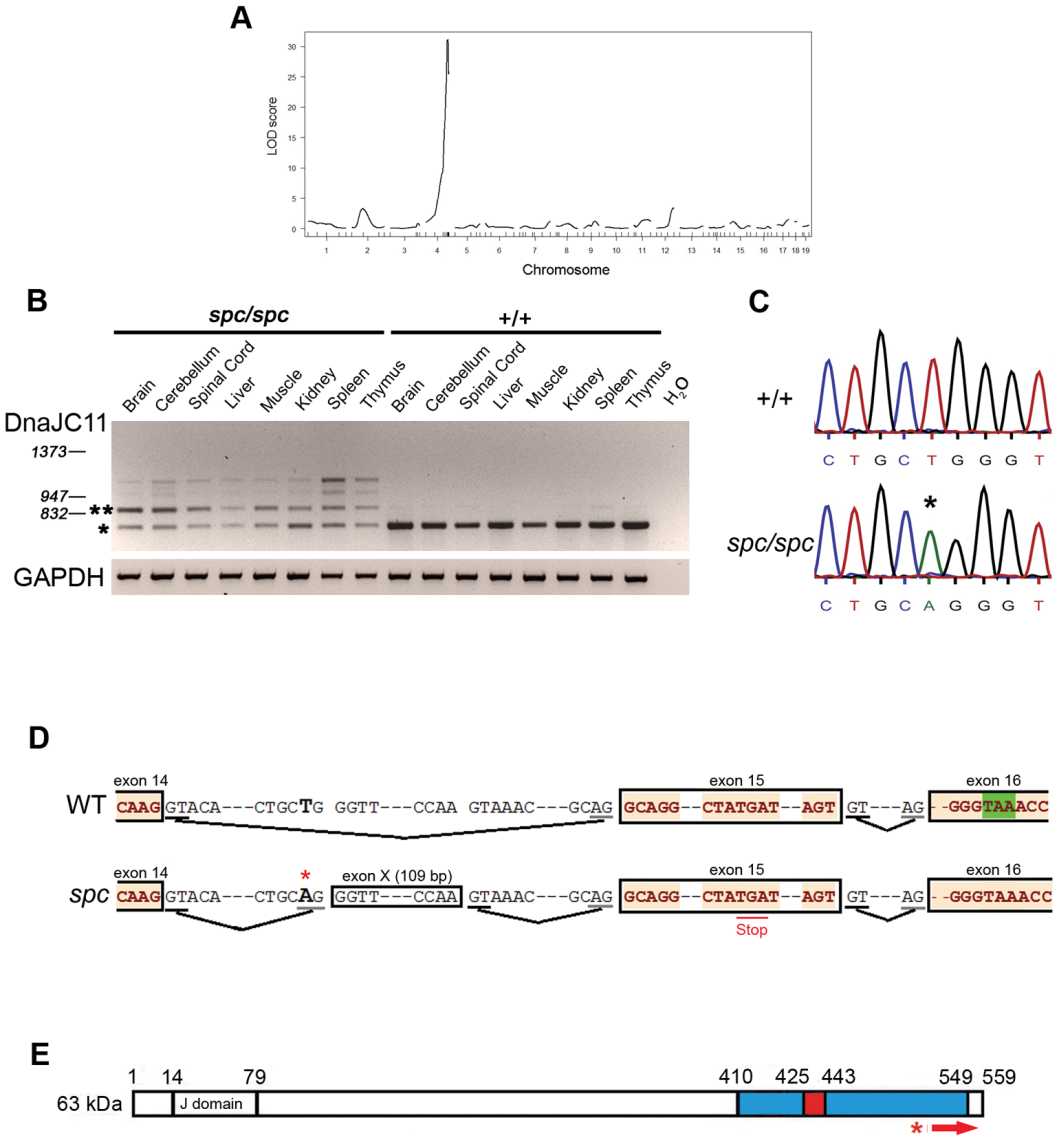
Mapping, identification and representation of the *spc* mutation. (A) Through genome-wide linkage analysis the causal mutation was mapped on distal chromosome 4. (B) Representative RT-PCR analysis on cDNAs from the indicated tissues of *spc/spc* and WT (+/+) littermate mice using a primer pair specific for the 3′- terminal coding region of DnaJC11 transcript. Numbers indicate base pair lengths of DNA marker. Single and double asterisk indicate the 735 bp WT and 844 bp mutant PRC products that were sequenced, respectively. A Gluceraldehyde 3-phosphate dehydrogenase (GAPDH) primer pair was used as a control for cDNA synthesis. Same results were observed in three more mice. (C) DNA sequencing of the *DnaJC11* gene in a WT control (+/+) and *spc/spc* littermate revealed an intronic T-to-A point mutation (asterisk). (D) Genomic organization of the *DnaJC11* gene within exons 14-16 and indicated splicing sites for the WT (upper) and the *spc* (lower) transcript. The T-to-A mutation is indicated with a red asterisk and it generates a splice acceptor AG site which results in the incorporation of an 109 bp intronic region (exon X) into the mature transcript. The insertion introduces a premature stop codon (red underlining). Green highlighted is the physiological WT stop codon. Black line, splice donor sites; Grey lines, splice acceptor sites. (E) Primary structure of the 63 kDa muDNAJC11 protein. J denotes the J domain, regions in blue and red represent the DUF3395 domain and the predicted coiled coil region, respectively. Numbers denote amino acid residues. Red asterisk denotes the site of the mutation at the protein level and the red arrow the stretch of the protein that is predicted to be mutated due to the frameshift.

To map the causal mutation, linkage analysis of 64 F2 mice (37 affected and 27 controls) was performed using a set of 83 polymorphic markers (SSLPs and SNPs) that spanned the whole genome except the sex chromosomes, considering that the trait was inherited in a recessive autosomal manner. Statistical analysis gave a logarithm of odds score of 31,1 for distal chromosome 4qE2 (Figure 4A). Another 842 meioses were analyzed for fine mapping in order to reduce the number of candidate genes, ending up with a genetic region of 2 Mbp between SNPs rs3665061 and rs32610416 (Table S1). This region, according to UCSC genome browser, contained 25 genes. Upon PCR analysis of the various cDNA fragments corresponding to these genes, additional splicing products were identified for the DnaJ (Hsp40) homologue, subfamily C, member 11 (*DnaJC11*) gene (ENSMUSG00000039768) in the mutant samples with distinct pattern in different tissues (Figure 4B). The primer pair that was used amplified products extending from exon 11 to the stop codon in exon 16 (Figure S2). The expected 735 bp long PCR product was also detected in the mutant samples (Figure 4B). Sequencing of the most abundant of these additional splicing products in the brain from *spc/spc* mice revealed a 109 bp long intronic insertion corresponding to a genomic region between exons 14 and 15. Sequencing of this intron and its splicing junctions at the DNA level revealed an intronic T>A transition (Figure 4C) that generated a splice acceptor site leading to the incorporation of the 109 bp intronic sequence (exon X) into the mature transcript (Figure 4D). This insertion changed the reading frame and was predicted to result in the replacement of the 51 C-terminal amino acids of the protein by 43 different ones (Figure S3). This novel mutated DNAJC11 protein product is predicted to have a molecular mass of 62 kDa, only 1 kDa less than the WT protein. This frame shift, however, is also predicted to generate a novel termination codon, which is located 107 nucleotides apart from the next exon-exon junction (Figure S2). According to the “50 nucleotide rule” this premature termination codon is predicted to render this mutant transcript subject to non-sense mediated decay [27].

DNAJC11 is a member of the J protein family also known as the Heat Shock Protein 40 (HSP40) family of co-chaperones. This family is defined by the presence of a highly conserved J domain and, based on the presence or absence of other known protein motifs, it is further subdivided into three classes (I, II and III or A, B and C) [28]. HSP40s are generally known to regulate HSP70-mediated ATP hydrolysis through their J domain, acting as co-chaperones, but their functional role is extremely diverse [28]. DNAJC11 does not have an assigned function yet. Multiple sequence alignment showed that it is a very highly conserved J protein in vertebrates (Figure S4) and invertebrates [29]. It has been reported to co-immunoprecipitate along with a number of mitochondrial proteins in human heart mitochondria and human HEK293 cells [19], [23]. Moreover, Pagliarini et al. detected mouse DNAJC11 (muDNAJC11) at high levels in mitochondria from all tissues examined using mass spectrometry [30]. According to Ensembl database the *muDnaJC11* gene is predicted to encode three protein isoforms of 63, 45 and 15 kDa (Figure S5). However, only the 63 kDa isoform is predicted to be affected at the protein level by the identified mutation (Figure S5).

In order to identify structural domains in muDNAJC11 we performed a bioinformatics analysis. Using the 63 kDa isoform sequence we found that the J domain is located at the N-terminus of the protein, between amino acid residues 14 and 79, and that there is also a Domain of Unknown Function (DUF3395) at the C-terminus of the protein, between amino acid residues 410 and 549 (Figure 4E). A coiled coil region was predicted to be located between residues 425 and 443 with 99% probability. Further *in silico* investigation, using homology molecular modelling, defined a reliable model for the N-terminal muDNAJC11 domain (Figure S6) and using protein secondary structure prediction techniques a suitable model was fitted for the DUF3395 domain (Figures S7-Figures S8). Structurally, this domain resembles the only structurally known C-terminal domain of the protein HSP40/MAS5/YDJ1 from *Saccharomyces cerevisae* (PDB code 1NLT) [31]. Such β-barrel domains are known to be involved in protein-protein associations to form multimers [32]. In the predicted frameshifted mutant protein the C-terminal domain bears no resemblance with the WT one and thus probably lacks the protein-protein association properties.

### Genetic Confirmation of the *DnaJC11* Mutation

**Figure 5:**
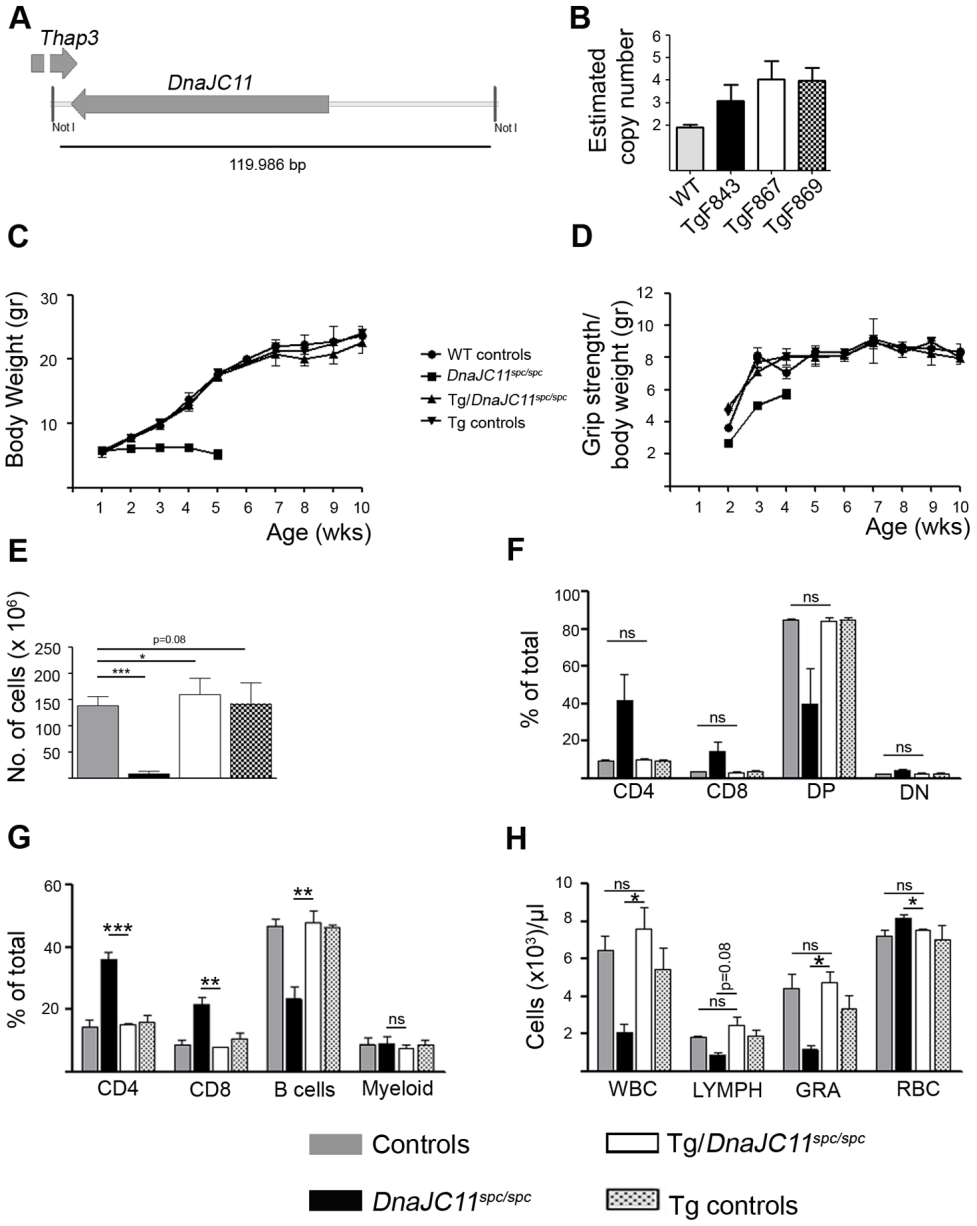
Complete rescue of the *DnaJC11^spc/spc^* phenotype through expression of the human *DnaJC11* gene. (A) Schematic representation of the human BAC clone fragment that was used for the generation of Tg*huDnaJC11* mice. Genes and their orientation are indicated as well as NotI sites that were used for digestion. Horizontal line and number below represent the fragment length. (B) Copy number determination, by qPCR, of three transgenic lines, TgF843, TgF867, and TgF869 (n = 5-9 per group) using a primer pair common for both mouse and human *DnaJC11* genes. WT mice were considered to carry 2 copies of *DnaJC11*. (C) Body weight and (D) grip strength (normalized to body weight) curves for the indicated genotypes. All mice used were sex matched littermates, (n = 8). (E) Rescue of the thymic hypoplasia shown as total thymic cellularity, (n = 3). (F) Restoration of thymic subpopulations distribution in rescued mice (n = 3) as determined by flow cytometry after staining with antibodies against CD4 and CD8. Statistical analysis between controls and rescued (Tg/*DnaJC11^spc/spc^*) mice is indicated. DP, double positive; DN, Double Negative. (G) Restoration of splenic subpopulations distribution in rescued mice. B cells and myeloid cells were defined as the ones positive for markers B220 and CD11b respectively, (n = 3). (H) Restoration of the leucopenia phenotype and the increased red blood cell phenotype in rescued mice (n = 3). Data represent means ± SE.

To confirm that the intronic point mutation identified in the *muDnaJC11* gene was indeed causal of the phenotype developed in the *spc/spc* (*DnaJC11^spc/spc^*) mice, genetic rescue experiments were performed by generating transgenic mice (Tg*huDnaJC11*) that carry the whole genomic region of human *DnaJC11* (*huDnaJC11*) and crossing them into the *DnaJC11^spc/spc^* background. As the human and mouse 63 kDa DNAJC11 proteins share 97% identity and 99% homology, they are expected to perform redundant functions. An approximately 120 kb genomic fragment containing the whole *huDnaJC11* gene was isolated from a bacterial artificial chromosome (BAC) clone (Figure 5A) and was used for pronuclear microinjections. The BAC fragment that was used contained also part of the *Thap3* gene but all the 5′-region of this gene up to the base encoding the first 89 amino acids of the respective protein was excluded. Thus, no protein from this gene is expected to be produced. Upon microinjections, seven transgenic founders were obtained, all of which were fertile and appeared healthy, exhibiting no obvious neurological symptoms. Out of the seven founders, three (TgF843, TgF867 and TgF869) were chosen to establish transgenic lines in order to use them in rescue experiments. Copy number determination by quantitative real-time PCR (qPCR) using a primer pair common for the human and mouse *DnaJC11* showed that all three transgenic lines carried one or two transgene copies (Figure 5B). qPCR for TgF869 line, which carried 2 copies of the transgene, verified a 2 to 6 fold increase of *DnaJC11* transcript levels depending on the tissue (Figure S9A). huDNAJC11 overexpression was also verified in Western blot analyses for many tissues (Figure S9B). Crossing each of the three transgenic lines into the *DnaJC11^spc/spc^* background completely rescued the premature lethality, growth retardation and the reduced grip strength displayed by the *DnaJC11^spc/spc^* mice (Figure 5C-D). No neurological symptoms manifested in these rescued lines within a period of one year. The TgF869 line was further investigated in rescue experiments. Indeed, in TgF869/*DnaJC11^spc/spc^* mice the motor neuron vacuolation phenotype and abnormal cristae structures (Figure S10A-B), the thymic and splenic hypoplasia, the thymic and splenic subpopulations phenotype, and the leucopenia were completely rescued (Figure 5E-H). These data genetically confirm the causal role of the *DnaJC11* mutation in the *DnaJC11^spc/spc^* phenotype and that the mouse and human genes have redundant functions.

### Mitochondrial Localization of the WT DNAJC11 Protein

**Figure 6:**
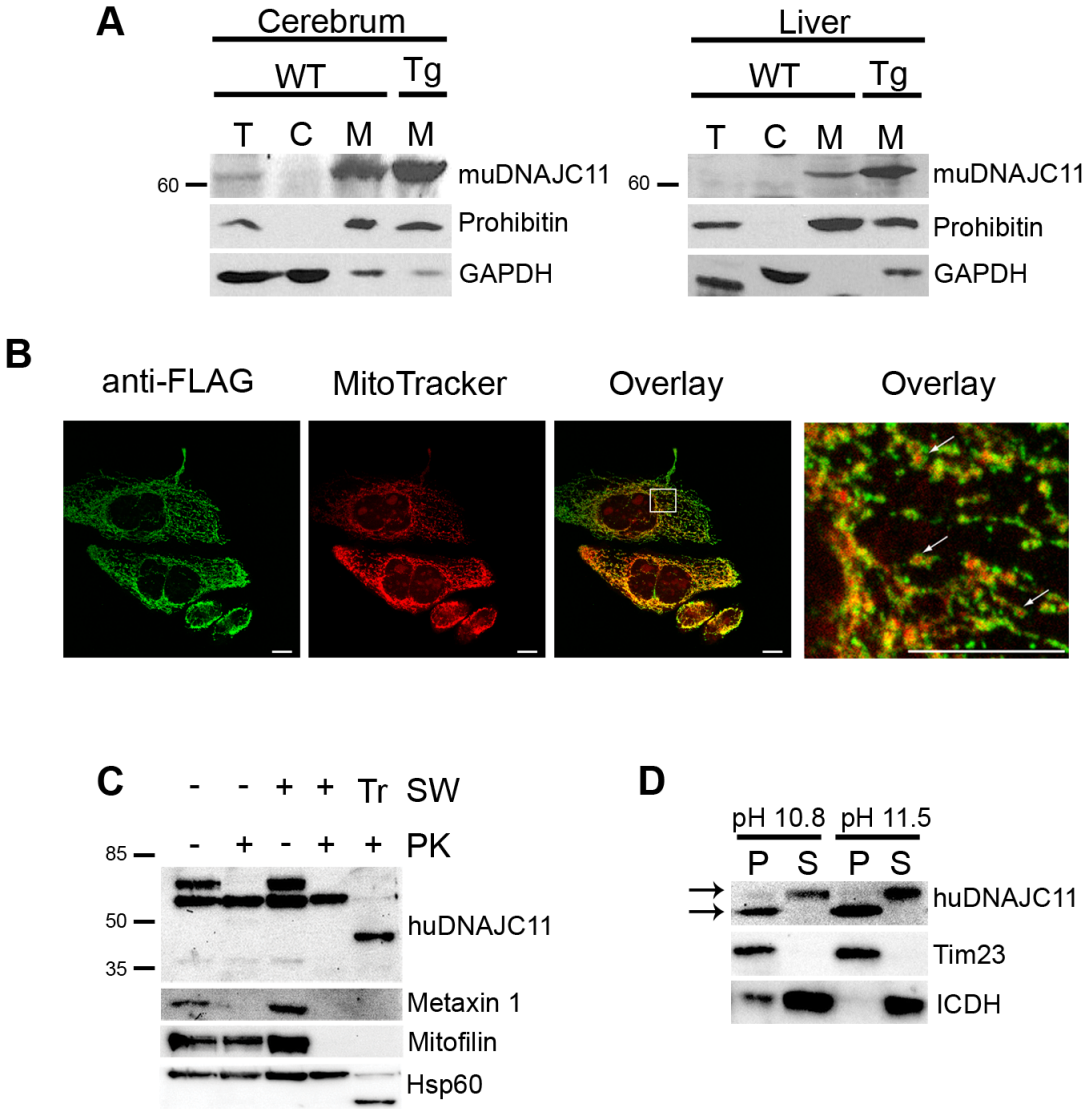
Mitochondrial and submitochondrial localization of DNAJC11. (A) Representative Western blot analysis on total protein RIPA extracts (T), on cytosolic (C) and mitochondrial (M) fractions of cerebrum and liver tissue from WT mice. The hu*DnaJC11* transgenic (Tg) samples served as positive controls. Prohibitin served as a mitochondrial specific marker and GAPDH as a cytoplasmic marker. (B) Fluorescence microscopy of HeLa cells transfected with a C-terminally FLAG-tagged huDNAJC11 cDNA of the 63 kDa isoform (green channel) and stained with the mitochondrial specific dye MitoTracker Orange (red channel). Scalebar, 100 µm. (C) Proteinase K protection assay on isolated mitochondria from HeLa cells. 50 µg of mitochondria were either subjected to swelling in the hypotonic buffer (SW, +) or were incubated in the isotonic buffer (SW, -) and then were treated with proteinase K (PK, +) or not (PK, -). A buffer containing 1% TritonX-100 (Tr) was used to solubilize all mitochondrial proteins and render them accessible to proteinase K. Samples were then analyzed by SDS-PAGE and Western blot with the indicated antibodies. Metaxin 1 is an outer membrane protein, mitofilin is an intermembrane space exposed inner membrane protein and Hsp60 is matrix localized. Numbers indicate molecular mass of protein marker in kDa. (D) Sodium carbonate extraction of isolated mitochondria from HeLa cells. Mitochondria were extracted under the two indicated pH conditions and the membranes were collected by ultracentrifugation. Pellet (P) and supernatant (S) were analyzed by SDS-PAGE and Western blot and probed with the indicated antibodies. Tim23 is an integral inner membrane protein and ICDH is a soluble matrix protein. Arrows indicate the 63 kDa isoform and the putative ∼57 kDa isoform. Tim23, Translocase of inner mitochondrial membrane 23 homolog; ICDH, isocitrate dehydrogenase.

In order to examine the subcellular localization of muDNAJC11, cerebrum from WT mice was processed to obtain either whole protein extract or cytosolic and mitochondrial fractions that were then subjected to SDS-PAGE and Western blot analysis, using an antibody against human and mouse DNAJC11. Our results showed that DNAJC11 migrated between the range of 60 and 70 kDa as expected (Figure 6A). Specificity of the antibody was also confirmed by increased levels of DNAJC11 in the Tg*huDnaJC11* mice (Figure 6A). No other isoforms could be verified in this way. DNAJC11 band was present in the mitochondrial but not in the cytosolic fraction and was hardly visible in the whole protein extract (Figure 6A). Similar results were obtained in liver protein extracts.

To verify whether the highly conserved 63 kDa huDNAJC1 isoform also displays mitochondrial localization, fluorescence microscopy was performed in HeLa cells transiently expressing FLAG-tagged huDNAJC11 using an anti-FLAG antibody. Our results showed that huDNAJC11 predominantly co-localized with MitoTracker Orange, which is a known mitochondrial marker (Figure 6B), confirming mitochondrial localization. Closer examination of residual FLAG-huDNAJC11 signal showed that even when not overlapping with MitoTracker, FLAG-huDNAJC11 was localized peripherally to MitoTracker, pointing towards an OM localization since MitoTracker is known to stain the mitochondrial matrix.

We next performed proteinase K protection assays and alkaline extraction experiments on mitochondria isolated from HeLa cells to define submitochondrial localization of huDNAJC11. According to Ensembl database, the *huDnaJC11* gene is predicted to encode 5 protein isoforms with a molecular mass of 63, 59, 57, 52 and 35 kDa respectively. In order to detect the predicted endogenous huDNAJC11 isoforms in Western blots we used a different antibody. With this antibody the biggest 63 kDa isoform could be detected consistently in isolated mitochondria from HeLa cells (Figure 6C). Interestingly, two additional lower bands were also visible but not consistently, with their detection depending on cell line clone and gel conditions and were usually detected in lower levels (Figure 6C). These bands migrated with a molecular mass of approximately 57 and 35 kDa, possibly representing two additional huDNAJC11 isoforms. Interestingly, our results showed that the various isoforms displayed different submitochondrial localizations. In the absence of mitochondrial swelling, the huDNAJC11 63 kDa isoform was accessible to proteinase K, suggesting localization of the protein in the OM, while the 57 kDa isoform was accessible to proteinase K only after the addition of Triton-X, suggesting mitochondrial matrix localization or an IM localization, with the large part of the protein being protease protected (Figure 6C). The smallest, 35 kDa isoform, behaved either as an IMS or an IM protein exposed to the IMS.

Alkaline extraction of mitochondria isolated from HeLa cells again showed differential association of huDNAJC11 isoforms with the different fractions. The 63 kDa isoform associated with the soluble fraction suggesting that the protein is peripherally associated with the OM and not anchored on or integrated within it (Figure 6D). The 57 kDa isoform was associated with the pellet fraction suggesting that this isoform could be anchored to the IM, facing the mitochondrial matrix. Due to its low abundance we were not able to detect the 35 kDa isoform in this assay.

### Analysis of Mutant muDNAJC11 Protein in Various Tissues and Biochemical Interaction of huDNAJC11 with Members of the MICOS Complex

**Figure 7:**
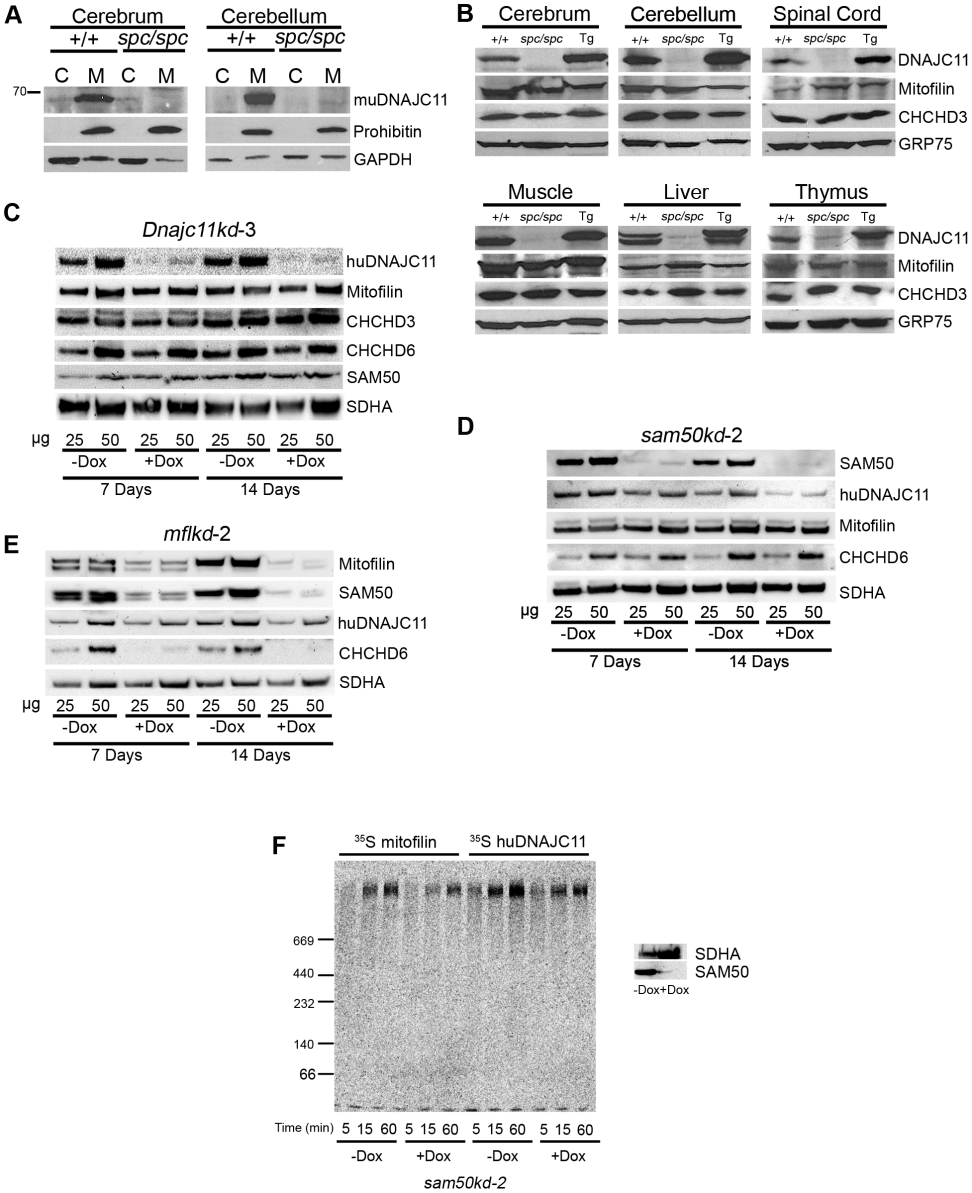
Expression analysis of mutant muDNAJC11 and biochemical interaction of huDNAJC11 with MICOS members. (A) Western blot analysis of fractionated brain tissue from *DnaJC11^spc/spc^* (*spc/spc*) mice and WT (+/+) littermates showing the loss of muDNAJC11 protein in *DnaJC11^spc/spc^* tissue. Prohibitin is a mitochondrial specific marker and GAPDH a cytoplasmic marker. (B) Equal amounts of isolated mitochondria from the indicated mouse tissues were analyzed by Western blot and probed for known members of the MICOS complex. Glucose related protein 75 (GRP75) was used as a loading control. (C) *dnajc11kd*-3, (D) *sam50kd*-2 or (E) *mflkd*-2 cells were grown in the absence (-Dox) or presence (+Dox) of doxycycline for 7 or 14 days, mitochondria were isolated, and 25 or 50 µg of protein were analyzed by SDS-PAGE and probed for the indicated proteins. SDHA, the component of the respiratory complex II, was used as a loading control. CHCHD3 and 6, coiled-coil-helix-coiled-coil-helix domain containing protein 3 and 6; SAM50, Sorting and assembly machinery 50; SDHA, Succinate Dehydrogenase subunit A. (F) Mitochondria from non-induced and induced *sam50kd-2* knockdown cells after 7 days of induction with doxycyclin (Dox) were isolated and incubated with the radiolabeled mitofilin and DNAJC11 (the longest isoform) for the indicated time periods. Samples were analyzed by BN-PAGE and autoradiography. The panel on the right hand side shows the control of the knockdown, where 50 µg of mitochondria from –Dox and +Dox samples were analyzed by SDS-PAGE and Western blot using antibodies against Sam50 and SDHA.

The predicted replacement of the last 51 amino acids of the muDNAJC11 by 43 different ones could interfere with proper mitochondrial localization of the protein. Thus, in order to examine the subcellular localization and the tissue distribution of the DNAJC11*^spc^* mutant protein, Western blot analyses on cytosolic and mitochondrial fractions were performed in various tissues. Our results showed that in cerebrum and cerebellum of *DnaJC11^spc/spc^* mice the muDNAJC11 protein was not present in either fraction (Figure 7A). Additional Western blots on isolated mitochondria from various tissues of *DnaJC11^spc/spc^* mice revealed that the *DnaJC11^spc^* mutation resulted in a reduction of muDNAJC11 protein levels ranging from severe to complete depletion depending on the tissue (Figure 7B). Thus, it is suggested that the *DnaJC11^spc^* allele ranges from severely hypomorphic to null depending on the tissue. The possibility that the observed muDNAJC11 bands could represent the predicted mutant form of the protein is open, though. The two proteins, WT and mutant, differ only in 1 kDa in mass and thus could not be resolved.

Two previous studies reported the co-immunoprecipitation of huDNAJC11 along with known MICOS complex proteins [19], [23]. As shown in the respective studies, depletion of the members of the MICOS complex such as mitofilin [18], CHCHD3 [20], CHCHD6 [22] or SAM50 [17] results in altered levels of other MICOS complex proteins. Thus, we investigated whether depletion or overexpression of DNAJC11 in our *DnaJC11^spc/spc^* and Tg*huDnaJC11* mice respectively resulted in altered levels of some of the core components of the MICOS complex, mitofilin and CHCHD3. Western blot analyses for these proteins showed that neither depletion nor overexpression of DNAJC11 influenced the levels of either mitofilin or CHCHD3 in the tissues examined (Figure 7B).

In order to examine the potential effect of huDNAJC11 depletion on the protein levels of other MICOS complex proteins, we generated doxycycline (Dox) inducible huDNAJC11 knockdown in HeLa cells, designated as *Dnajc11kd-3*. Of note, in this cell line only the longest two huDNAJC11 isoforms, 63 kDa and 59 kDa, were targeted by the shRNA. However, only the 63 kDa isoform could be verified to be downregulated upon Dox induction as the rest isoforms could not be detected consistently in Western blots. Depletion of huDNAJC11 after 7 or 14 days of Dox application was almost complete (Figure 7C). Nevertheless and consistent with our findings in mouse tissues none of the other MICOS complex protein levels (mitofilin, CHCHD3, CHCHD6 and SAM50) were affected (Figure 7C).

To investigate whether depletion of mitofilin or SAM50 affects the levels of huDNAJC11, we utilized a previously described [17] Dox inducible human mitofilin (*mflkd-2*) and SAM50 (*sam50kd-2*) HeLa-derived knockdown cell line. After 7 and 14 days of Dox application both mitofilin and SAM50 were severely depleted and at the same time DNAJC11 protein levels were observed to be mildly but consistently reduced (Figure 7D-E). In the SAM50 depleted cells other members of the MICOS complex, such as mitofilin and CHCHD6 were not affected suggesting a more direct role of SAM50 in the sustainment of DNAJC11 protein levels (Figure 7D). In the mitofilin depleted cells, though, other members of the MICOS complex like CHCHD6 (Figure 7E), SAM50 and CHCHD3 [17] were affected. These findings support indirecty an interaction of the established MICOS complex proteins mitofilin and SAM50 with DNAJC11 in human cells.

In order to examine whether DNAJC11 exists in the same complex with mitofilin and SAM50, we imported radiolabeled mitofilin and 63 kDa huDNAJC11 into mitochondria isolated from inducible SAM50 HeLa knockdown cells. Radiolabeled mitofilin and DNAJC11 was found to assemble in a time-dependent manner into a large complex of >700 kDa that resembles the complex described previously as the mitochondrial intermembrane space bridging (MIB) complex where mitofilin, SAM50 and CHCHD3 co-exist [17]. Interestingly, the depletion of SAM50 reduced the assembly of this complex (Figure 7F), as in the case of imported CHCHD3 [17]. These results strongly support the presence of DNAJC11, SAM50, mitofilin and CHCHD3 in the same mitochondrial complex.

## Discussion

Using random ENU mutagenesis in the mouse, we have identified an autosomal recessive phenotype comprising pronounced motor dysfunction and blood/lymphoid abnormalities, caused by a splicing point mutation in a novel mitochondrial protein, DNAJC11. Light and transmission electron microscopic observations of the spinal cord of mutant mice (*DnaJC11^spc/spc^*) showed severe vacuolation of motor neurons in the ventral horn with the next most affected CNS region being the medulla. This type and pattern of vacuolation is reminiscent of the one observed in two mouse models of familial ALS expressing mutant Superoxide Dismutase 1 (SOD1) alleles [33], [34], [35]. In those models, like in *DnaJC11^spc/spc^* mice, the vacuoles have dual origin, the endoplasmic reticulum and mitochondria. It would be tempting to hypothesize a possible common mechanism of motor neuron pathology between the mutant SOD1 models and *DnaJC11^spc/spc^* mice, or two different ones converging in mitochondrial structure and dysfunction. However, the identified pathology does not necessarily mean dysfunction of motor neurons which surely needs to be investigated in the future. At any rate, the manifestation of CNS and especially motor neuron specific pathology due to a mutation in *DnaJC11* surely opens new possibilities for the search of novel contributors in neuromuscular diseases, particularly in conditions with motor neuron involvement like ALS, and especially in the light of our finding that expression of huDNAJC11 can fully compensate the depletion of muDNAC11.

DNAJC11 is a member of the J protein family (Hsp40s) of co-chaperones that are known to interact with Hsp70 proteins, regulating their ATP hydrolysis function and thus facilitating a wealth of cellular processes [28], [36], [37]. These include binding to non-native or nascent polypeptides, multiprotein complex assembly and disassembly, translocation of proteins across organelle membranes and probably others. The list of mouse mitochondrial proteins compiled by Pagliarini et al. [30] revealed five mitochondrial J proteins, four of which are classified in the C subclass (DNAJC4, 11, 15, 19) and one in the subclass A (DNAJA3). Of note, mutations in *DnaJC19*, probably a member of the TIM complex, have been reported to cause dilated cardiomyopathy with ataxia, a Barth-like syndrome [38], [39]. Many others, non-mitochondrial J protein mutations give rise to neurological or neuromuscular phenotypes. *DnaJA3* knockout mice develop dilated cardiomyopathy [40], a mutation in *DnaJB6* cause autosomal dominant myopathy [41] and mutations in *DnaJC5* and *9* cause or are associated with autosomal-dominant adult-onset neuronal ceroid lipofuscinosis and schizophrenia with deficits in attention, respectively [42], [43], while *DnaJC29* (also known as sacsin) is mutated in autosomal recessive spastic ataxia of Charlevoix-Saguenay [44]. These phenotypes highlight the functional importance of J proteins in proper nervous system function.

DNAJC11 in animals does not have an assigned function yet. It does not have a yeast homologue but is very highly conserved in the animal kingdom and is also present in plants [29], [45], highlighting its fundamental importance for cell function. In *Arabidospis thaliana,* DNAJC11 homologue, OWL1 (*atDjC28* or *At2g35720*) is also ubiquitously expressed but displays a dual nuclear and cytoplasmic localization and has also been shown to be involved in the very low light fluence response [46]. In the same study, direct interaction of OWL1 with a nuclear transcription factor is reported although additional functions are also speculated. It is highly unlikely that animal DNAJC11 has a similar nuclear function since it does not show any nuclear localization as confirmed by our confocal analysis (Figure 6B). Our bioinformatics analysis showed that the full length 63 kDa isoform of DNAJC11, apart from the J domain which is located at the N-terminus of the protein, contains a Domain of Unknown Function (DUF3395) at the C-terminus region possibly involved in protein-protein interactions for multimers formation.

The identified *DnaJC11* mutation and its effects at the mRNA and protein levels are of particular interest. Interestingly, only the 63 kDa isoform is predicted to be affected at the protein level by the identified mutation. The novel generated splice site affects splicing events in the region profoundly, giving rise to a wealth of transcripts through a mechanism that requires further investigation. We were able to sequence the most abundant of these transcripts which is predicted to encode a C-terminal frameshifted product, which probably lacks the protein-protein association properties. Although the mutant, as well as the WT transcripts are expressed, the reduction of the DNAJC11 protein levels is almost complete in most tissues, suggesting a post-transcriptional regulatory mechanism that affects both transcripts. With regard to the sequenced mutant transcript, non-sense mediated decay offers such an explanation since this transcript meets the “50 nucleotide rule” [27], although the transcript levels reduction is far from complete. Importantly, all aspects of this novel phenotype were rescued through the expression of the human ortholog in transgenic mice highlighting the redundant functions of the human and mouse *DnaJC11* genes which share 97% identity.

Two groups have reported the co-immunoprecipitation of huDNAJC11 along with members of the MICOS complex [19], [23], which is involved in the formation of mitochondrial cristae [14], [15], [16], [17], suggesting a possible interaction of DNAJC11 with MICOS proteins. Our importation experiments also strongly support the presence of DNAJC11, SAM50, and mitofilin in the same mitochondrial complex. Jans et al., using super-resolution microscopy, very elegantly showed that huDNAJC11 does not display the characteristic submitochondrial distribution and positioning shared by the MICOS core components but had a more patchy distribution, proposing that only a subset of huDNAJC11 molecules is in physical contact with core MICOS proteins [47]. The mitochondrial morphology phenotype seen in our *DnaJC11^spc/spc^* mice and the fact that depletion of mitofilin and SAM50 affect the levels of DNAJC11 in HeLa cells strengthen its candidacy as an additional MICOS protein or MICOS interactor having a peripheral role in MICOS function, maybe participating or mediating the interactions of MICOS with one of its numerous interactor complexes in the OM like the TOM or the SAM complex. This hypothesis is based on our submitochondrial localization data that assign to the 63 kDa DNAJC11 isoform a peripheral OM topology. Moreover, possible functional interactions between DNAJC11 and Hsp70 chaperons have to be investigated.

Depletion of specific MICOS proteins has been shown to result in the destabilization of the MICOS complex and reduction of the levels of other MICOS proteins [16], [17], [18], [20]. Because of its co-chaperone nature we speculated that DNAJC11 could function as a recruitment or stability factor for other MICOS proteins. Surprisingly, the muDNAJC11 depletion observed in all tissues of *DnaJC11^spc/spc^* mice did not alter the levels of mitofilin or CHCHD3 leaving the question of whether there is indeed a functional relationship between DNAJC11 and the MICOS complex unanswered. Other MICOS proteins that we did not check could be affected by the loss of DNAJC11. Another possibility is that although the levels of these two MICOS proteins were not affected, the general stability of the MICOS complex could be compromised. Harner et al. showed that upon deletion of every single member of the yeast MICOS complex not all other MICOS proteins levels were affected, but in each case the complex fell apart [14]. Thus, further characterization of the MICOS complex in WT and *DnaJC11^spc/spc^* mice is required in order to elucidate whether muDNAJC11 interacts with the MICOS complex in mouse tissue and in order to clarify a possible connection between this putative interaction and *DnaJC11^spc/spc^* mice pathology.

Identification of possible interacting partners and thus elucidation of the function of DNAJC11 is complicated by the presence of multiple isoforms. In mouse tissues, out of the 3 predicted muDNAJC11 isoforms, we were able to identify in Western blots the longest 63 kDa one, which we found to be ubiquitously expressed not only in the central nervous system but also in extraneuronal tissues. In HeLa cells, out of the 5 predicted huDNAJC11 isoforms we could detect the 63 kDa and two additional bands of approximately 57 and 35 kDa that could represent two additional isoforms. Of these only the 63 kDa one could be validated by its downregulation in *dnajc11kd-3* cell lines. Of note, human isoforms showed three different submitochondrial localizations; peripheral OM localization for the 63 kDa isoform, matrix or IM localization with possible membrane anchoring for the 57 kDa isoform and IMS or IM protein localization for the smallest 35 kDa isoform. Such different topology of the DNAJC11 isoforms increases the number of possible interacting partners and thus functions of DNAJC11. Dual localization of MICOS proteins is not something new. Darshi et al. showed that CHCHD3 is enriched both in OM and IM fractions but with differential abundance [20]. This dual localization of CHCHD3 is suggested to have to do with participation in two different “versions” or species of the MICOS complex itself. Independent investigators have suggested the existence of a holo-complex with a reported molecular mass of approximately 1,2 to 1,5 MDa, that probably also involves interactions with OM proteins, and a smaller complex with a molecular mass of approximately 0,7 MDa [14], [17], [18]. Different isoforms of DNAJC11 could interact with different complexes in a similar way. Again, identification of specific protein interactors is necessary.

The reported *DnaJC11* mouse mutant is one of the few mouse mutants with mutations in proteins involved in mitochondrial morphology. Other mouse models of this category are mutants for Optic Atrophy 1 (OPA1) [49], [50], [51], which is a well studied protein of the IMS involved in mitochondrial fusion, cristae remodeling and apoptosis [52], [53], and which has been reported to interact with CHCHD3 [20]. The neuronal subpopulation-specific and blood/lymphoid pathology of the *DnaJC11^spc^* ^/*spc*^ mice raises very interesting questions about the *in vivo* role of the DNAJC11 protein. In depth characterization of the *DnaJC11^spc^* ^/*spc*^ mice at the molecular, biochemical, cellular and ultrastructural level could contribute to the elucidation of the physiological role of *DnaJC11* and its involvement in disease pathogenesis. Finally, our study paves the way for the identification of polymorphisms and functional mutations in *DnaJC11* in patients with neuromuscular diseases.

## Materials and Methods

### Mouse husbandry

DBA/2J mice were purchased from the Jackson Laboratories. Mice were maintained and bred under specific pathogen-free conditions in the animal facility of Biomedical Sciences Research Center (BSRC) ‘Alexander Fleming’. Mice were sacrificed via CO_2_ inhalation followed by cervical dislocation.

### Ethics statement

All animal procedures were carried out in strict accordance to the Hellenic License for Animal Experimentation at the Biomedical Sciences Research Center ‘Alexander Fleming’ (Licence Protocol Number 1167/08.03.2012) issued after protocol approval by the Institutional Animal Ethical Committee of BSRC ‘Alexander Fleming’ (Protocol Number 258/13.02.2012)**.**

### ENU mutagenesis

G0 males of a mixed C57BL/6Jx129S6 background were treated with ENU (Sigma-Aldrich, Inc.) administered in three weekly doses at 100 mg/kg of body weight [24], [54]. Each G0 mouse was crossed to WT C57BL/6Jx129S6 females to produce G1 males that were further mated with WT females to produce G2 daughters that were subsequently backcrossed with the G1 parent to generate G3 progeny. These were screened phenotypically for recessive mutations. ENU mutagenesis was performed at BSRC ‘Alexander Fleming’. Mutant G3 mice were crossed at least 10 times into C57BL/6J background to obtain congenic N10 generation mice carrying the DnaJC11 mutation. All experiments presented were performed using at least N10 congenic mice.

### Grip Strength

The Grip Strength Test Meter, Model GS3 (Bioseb) was used. Mice were allowed to stand with four paws on the metal grid and were gently pulled by the tail until they were off the grid. The mean from three repeats was calculated for every measurement.

### Histopathological analysis

Tissue was dissected and fixed in 4% (M/V) formaldehyde (VWR) at 4°C overnight and then transferred in PBS before processing for paraffin embedding. Sections of 4 µm thickness were collected and processed for staining with hematoxylin/eosin.

### Neuropathological analysis and Transmission Electron Microscopy

Mice were sacrificed and transcardially perfused with 5 ml of ice cold fixative (3% electron microscopy grade glutaraldehyde in 0,1 M phosphate buffer). Tissue was dissected and kept in ice cold fixative while trimming into ∼1mm cubes. Samples were postfixed in the same fixative at 4°C overnight and were then washed in phosphate buffer, fixed with 1% OsO_4_ at room temperature, dehydrated through a graded series of alcohols, placed for 1 h in propylene oxide (Aldrich Chemistry) and left overnight in a propylene oxide/spurr resin 1∶1 solution. Samples were placed in fresh resin blocks and incubated >36 hours at 60°C for resin polymerization.

Semi-thin sections (1 µm thick) were obtained using an ultramicrotome, placed on glass slides, stained with 0,5% toluidine blue and photographed using an Olympus BX40 digital camera. Desired regions were identified and ultrathin (60–90 nm) sections were obtained, collected on copper grids, stained with uranyl acetate and lead citrate and observed under a JEOL JEM-1005 transmission microscope. Photos were taken with an Olympus DP71 digital camera.

### Flow Cytometry

Mice were sacrificed and thymus and spleen were quickly excised and ground in 5 ml ice cold PBS using a flat end glass pestle and size 40 mesh discs (Sigma-Aldrich, Inc.). Cell suspension was filtered through a 70 µm sheet and cells were pelleted by centrifugation. Thymocytes were resuspended in PBS and counted using an hematocytometer. Splenocytes were resuspended in 1,5 ml Gey's solution, pelleted by centrifugation, resuspended in PBS and counted. 3×10^6^ thymocytes per well were stained with CD4-Alexa700, CD8-Alexa647, CD25-PE, CD44-FITC. 1×10^6^ splenocytes per well were stained with CD4-Alexa700, CD8-Alexa647, B220-PerCP, Gr1-FITC and CD11b-PE. Antibodies were purchased from Biolegend.

### Biochemical markers and blood analysis

Clinical chemistry on serum samples and blood counts on peripheral blood samples, obtained by heart puncture, were performed by a commercial provider (Microanalysi SA, Athens) as previously described [55].

### Mapping and Sequencing

Heterozygous +/*spc* mice were outcrossed with DBA/2J mice. F1 mice were intercrossed and tail DNA from the resulting F2 mice was used for the genetic linkage analysis. SSLPs were analyzed on 4% high resolution agarose gels (Sigma-Aldrich, Inc.) and SNPs were analyzed by pyrosequencing using the Pyromark ID instrument (Biotage AB). A standard genome scan was conducted using the qtl library of R (The R Foundation for Statistical Computing, version 2.8.0) [56]. Log-likelihood linkage for single-trait analysis was established by non-parametric interval mapping of a binary model (diseased versus healthy control siblings), on 124 F2 animals in total, computed at 1 cM increments over the entire genome. Sequencing was carried out on PCR products, purified after agarose gel electrophoresis, as a service by MWG Biotech AG.

For *DnaJC11* genotyping the following primers were used for pyrosequencing on tail DNA: F: 5′-CCT CAG TGC CAG CAA AGT CT-3′, R, 5′-ACG GCC ACA GCC ACA GAT-3′ and seq, 5′-GCC TCT TTC TGG AAC C-3′.

### Generation and screening of transgenic mice

Human BAC clone RP11-262K21 was purchased from imaGenes (clone RPCIB753K21262Q). Isolated BAC DNA was digested with NotI and products were analyzed overnight by Pulsed Field Electrophoresis. The ∼120 kb product containing the human DnaJC11 gene was excised and and subsequently analyzed through a 4% low melting agarose gel before isolation with β-agarase (New England Biolabs) digestion. Fertilized F2 (C57BL/6J x CBA/J) oocytes were microinjected as previously described [57], [58] Microinjections and embryo implantations were carried out in BSRC 'Alexander Fleming‘ Transgenics Facility.

Screening of transgenic animals was performed by PCR analysis on tail DNA using the following primers: F, 5′-ACT CAT GGT TTG GGG TCC TT-3′; R, 5′-CTG GTT TTG TCT TCC CTC CA-3′, which hybridize on an intronic sequence of the human but not the mouse gene.

### Transgene copy number determination

Determination of transgene copy number was performed by qPCR using tail DNA from Tg*huDnaJC11* mice and WT littermates. A primer pair specific for both mouse and human *DnaJC11* gene was used: F, 5'-CCT CCG TGA GGA TGA GCT T-3'; R, 5'-GTC AAT GAC AAG AGC AGG AAG-3' The nuclear gene *HuR* was used as internal control for each sample: F, 5′-AGG ACA CAG CTT GGG CTA CG-3′ and R, 5′-CGT TCA GTG TGC TGA TTG CT. Standard curves were generated for both primer pairs and C_T_ values were determined and analyzed by the comparative C_T_ method. Fold change of transgenic samples compared to the WT was used to extrapolate the copy number for each transgenic line.

### Reverse Transcription and Real Time PCR (qPCR) analysis

Total RNA was extracted by the guanidinium thiocyanate phenol-chloroform method using Tri-Reagent (MRC Inc.). Concentration and quality of RNA was determined with a NanoDrop ND-1000 spectrophotometer.

2 µg of total RNA were treated with DNase1 amplification grade (Sigma-Aldrich, Inc.) to remove residual DNA, and they were reverse transcribed with M-MLV reverse transcriptase (Sigma-Aldrich, Inc.) using 0,5 µg of oligo d(T)_18_ primer (New England Biolabs). Reverse Transcription PCR was performed with the following primer pairs: *DnaJC11*, F, 5′-GGC CAG TCA GAC CTA CTT C-3′ and R, 5′-AGC TAG GCT GTC TGC ATG-3′; GAPDH, F, 5'-AGC ACC CCT GGC CAA GG-3' and R, 5'-CTT ACT CCT TGG AGG CCA TG-3'.

qPCR on cDNAs was performed using SsoFast EVA Green Supermix (Bio-Rad) on a Rotor-Gene 6000 RT-PCR machine (Corbett Life Science). Standard curves were generated for *DnaJC11* and for the β2-microglobulin (*B2M*), which was used as internal control. C_T_ values were determined and data were analyzed by the comparative C_T_ method. Samples were run in duplicates. The following primers were used: common for human and mouse *DnaJC11*, F, 5'-CAA AGG GAT GGG GAG AGT TG-3' and R, 5'-CGG GAT GAA AAC TGC AGA G-3'; *B2M*, F, 5'-TTC TGG TGC TTG TCT CAC TGA-3' and R, 5'-CAG TAT GTT CGG CTT CCC ATT C-3'.

### Tissue mitochondria isolation

Tissue mitochondria were isolated by differential centrifugation. After organ extraction, approximately 100 mg of tissue was homogenized manually in Mitochondria Isolation Buffer (MIB) (320 mM sucrose, 1 mM EDTA, 10 mM Tris, pH adjusted to 7,4) complemented with proteinase inhibitors cocktail (Roche) on ice, using a 1 ml glass mortar and pestle. Homogenate was centrifuged at 3000 g for 5 min, the supernatant was kept on ice and pellet was resuspended in MIB and centrifuged again at 3000 g for 5 min. The two supernatants were mixed and centrifuged at 12000 g for 10min to acquire the mitochondrial pellet and the cytoplasmic fraction. Protein concentration was determined with the Bradford method (Bio-Rad Protein Assay).

### Knock-down cell lines and isolation of mitochondria

Parental HeLa cells were purchased from ATCC (American Type Culture Collection, Manassas, VA, USA). HeLa cells with an inducible shRNA-mediated knockdown were generated as already described [59], [60]. The sequence of *dnajc11kd-3* shRNA was 5′- GCACCGTCTGATCATCAAACC -3′. shRNAs of *sam50kd-2* and *mflkd-2* were previously described [17], [59]. Single cell clones were isolated for each shRNA. Cells were cultivated in RPMI 1640 or DMEM (Gibco) supplemented with 10% FCS (Biochrom) and penicillin/streptomycin. Expression of shRNAs was induced by cultivating cells in media additionally containing 1 µg/ml doxycycline (BD Biosciences) for indicated time periods. Isolation of mitochondria was performed as described [59].

### Fluorescence Microscopy

Cells grown on glass coverslips were transfected with a pCDNA3 (Invitrogen) plasmid containing human *DnaJC11* cDNA fused to a carboxy-terminal FLAG-tag using Lipofectamine 2000 (Life Technologies). After 24 h to 36 h of expression, cells were stained by incubation with 150 nM MitoTracker Orange (Molecular Probes) in cell culture media for 30 min at 37°C. Samples were washed with PBS, fixed in 3,7% PFA and stained with an anti-FLAG antibody and the corresponding secondary fluorophore-coupled antibody. Samples were analyzed with a Leica confocal microscope using TCS software.

### Swelling and alkaline extraction

Alkaline extraction using 100 mM Na_2_CO_3_, pH 10,8 or pH 11,5 were performed as previously described [59], [61], [62]. For the swelling experiments and opening of the OM, freshly prepared mitochondria were incubated in isotonic (250 mM sucrose, 1 mM EDTA, 10 mM Tris, pH 7,6) or hypotonic (1 mM EDTA, 10 mM Tris, pH 7,6) buffer. Mitochondria were then treated with 50 µg/ml of proteinase K, inhibited later by addition of 2 mM PMSF. Additional sample of mitochondria was solubilized in 1% Triton X-100 and, after a 12000g spin to remove insoluble material, the supernatant was treated with proteinase K as already described.

### Protein import

*sam50kd-2* cell line and import into isolated mitochondria have already been described [59]. In short, *sam50kd-2* cells were grown for seven days in the presence of doxycyclin to induce the production of shRNA and Sam50 knockdown. Mitochondria from non-induced and induced knockdown cells were isolated and incubated with the radiolabeled Mitofilin and DnaJC11 (the longest isoform) at 37°C in the potassium-acetate import buffer (250 mM sucrose, 5 mM Mg-acetate, 80 mM Kacetate,20 mM Hepes, pH 7.4, 10 mM Na-succinate,1 mM ATP, and 1 mM DTT). Samples were then solubilized in the presence of 1% digitonin and analyzed by BN-PAGE as described [59].

### Antibodies

DNAJC11 antibody for HeLa cells experiments was purchased from Abnova and for mouse tissue from Proteintech; Mitofilin, CHCHD6 and CHCHD3 from Abcam; SDHA from Invitrogen; Hsp60 from Stressgen; Tim23 from BD Transduction Laboratories; Grp75 and GAPDH from SantaCruz Biotechnology; Prohibitin from NeoMarkers; ICDH from Biogenesis; SAM50 and Metaxin antibodies were raised in rabbits against a full-length 10xHis-tagged protein.

### Bioinformatic analysis

Primary structure of muDNAJC11 was determined using the search tool against the Pfam database (http://pfam.sanger.ac.uk/). Secondary structure prediction and alignment was performed using JPred [63]. Sequence homology searches were performed using BLASTP on both the non-redundand GenBank database and the SwissProt databases. Defining structural homology was performed using BLASTP on the sequences of the ProteinDataBank (PDB) database [64], [65], and the SwissModeller server [66]. Multiple sequence alignment for vertebrate DNAJC11 homologues was performed with ClustalX software. The coiled coil region was determined using the MARCOIL coiled-coil prediction software [67].

### Statistical analysis

Student's two tailed unpaired t test was performed using Prism 5 (GraphPad Software). p values were symbolized as follows: *p<0,05; **p<0,01; ***p<0,001; ns, p>0,05.

## Supporting Information

Figure S1 **Abnormal mitochondrial structure in** ***spc/spc*** **spinal cord motor neurons.** Representative electron micrographs of mitochondria (M) in motor neuron cell bodies from WT (a) and *spc/spc* mice (b-d). Asterisks indicate abnormally stacked or concentric membranes. Scalebar: 200 nm.
(TIF)

Figure S2 **Exon organization and primer pair positions of the sequenced** ***DnaJC11*** **brain transcripts.** Horizontal arrows denote the primer pair that was used for sequencing. Black horizontal lines denote the 735 and 844 base pairs PCR product from a wild type (WT) and a *spastic* mouse. The inserted 109 bp long additional exon in the *spastic* transcript is indicated with X. Stop codons of the two transcripts are indicated (TAA in WT, TGA in *spastic*). The 107 base pairs distance of the novel TGA stop codon in the *spastic* transcript from its next downstream exon-exon junction is also indicated.
(TIF)

Figure S3 **Protein sequence alignment of the C-terminal region of the WT and predicted mutant (Spastic) DNAJC11.** Grey bars denote percentage of conservancy. Asterisks denote 100% conservancy. Black arrow denotes the position of the first frameshifted amino acid of the predicted mutant protein.
(TIF)

Figure S4 **Multiple sequence alignment of the DNAJC11 63 kDa isoforms between the indicated vertebrate species.** Sequences were obtained from Ensembl database. Grey bars denote percentage of conservancy. Asterisks denote 100% conservancy.
(TIF)

Figure S5 **Schematic diagram of the three predicted isoforms of muDNAJC11.** Amino acid numbers for all recognized domains are shown. Region in blue represents the DUF3395 domain. Region in red represents the coiled coil domain. Red asterisk and red arrow represent the site of the mutation and the stretch of the predicted mutated sequence respectively. Yellow region in the 45 kDa isoform represents a region absent in the other isoforms. Black lines denote the protein regions of the 63 kDa isoform which are absent in the other isoforms.
(TIF)

Figure S6 **A structural model of the J domain of the N-terminal 14–72 amino acid residues of DNAJC11**. A-helices are represented as cyan springs and non ordered linkers as purple tubes.
(TIF)

Figure S7 **The secondary structure prediction for the C-terminal region of the 63 kDa isoform.** Each block runs horizontally along the protein's amino acid sequence and vertically indicating homologues sequence analysis, sequence conservation (boxes), and predicted secondary structure elements (α- helices as purple rods and β-strands as yellow arrows). Query, *Mus musculus*; Q7QDV2, *Anopheles gambiae*; B4J6Z1, *Drosophila grimshawi*; B3S076, *Trichoplax adhaerens*; Q9NVH, *Homo sapiens*; A9RS49, *Physcomitrella patens*; A8QCS0, *Malassezia globosa*; Q54PV9, *Dictyostelium discoideum*; B0D6Q8, *Laccaria bicolor*; C1E8E4, *Micromonas sp*.; A9PH24, *Populus trichocarpa*; A2QUD0, *Aspergillus niger*.
(TIF)

Figure S8 **A structural model of the C-terminal domain of the protein MAS5/HSP40/YDJ1.** This model resembles the C-terminal domain of the 63 kDa isoform of muDNAJC11 that may form the association domain. Α-helices are shown in cyan, β-strands are shown in purple. On the left, part of the coiled coil region is shown, represented as pink tube.
(TIF)

Figure S9 **Expression analysis of** ***DnaJC11*** **in TgF869 transgenic mice.** (A) qPCR analysis of *DnaJC11* expression using a primer pair common for both mouse and human *DnaJC11* transcripts. Cerebrum, Wt (n = 5), Tg (n = 8); Spinal Cord, WT (n = 3), Tg (n = 6); Muscle (n = 4); Thymus (n = 2-3). Data represent mean ± SE. (B) Western blot analysis of isolated mitochondria for DNAJC11 expression in various tissues of WT and Tg mice. Grp75 served as a loading control. Student's t test was performed for statistical analysis. ***p< 0.001, **p<0.01, *p<0.05.
(TIF)

Figure S10 **Normal motor neuron morphology in rescued (TgF869/** ***DnaJC11^spc/spc^*** **) mice.** (A) Representative toluidine blue stained semi-thin resin sections showing motor neurons in the ventral horn of the spinal cord from two, 2 month old rescued mice. Motor neurons of rescued mice (n = 2) had a perfectly normal appearance and were indistinguishable from the WT ones. Scalebar: 20 µm. (B) Representative electron micrographs of mitochondria in motor neuron cell bodies from rescued mice. Scalebar: 200 nm.
(TIF)

Table S1 **Fine mapping results.**
(DOC)

Video S1 (AVI)
