# Supplementary figures and images for: A Splicing Mutation in the Novel Mitochondrial Protein DNAJC11 Causes Motor Neuron Pathology Associated with Cristae Disorganization, and Lymphoid Abnormalities in Mice
Source: PLoS One. 2014 Aug 11;9(8):e104237. doi: 10.1371/journal.pone.0104237 (PMC4128653; doi:10.1371/journal.pone.0104237)

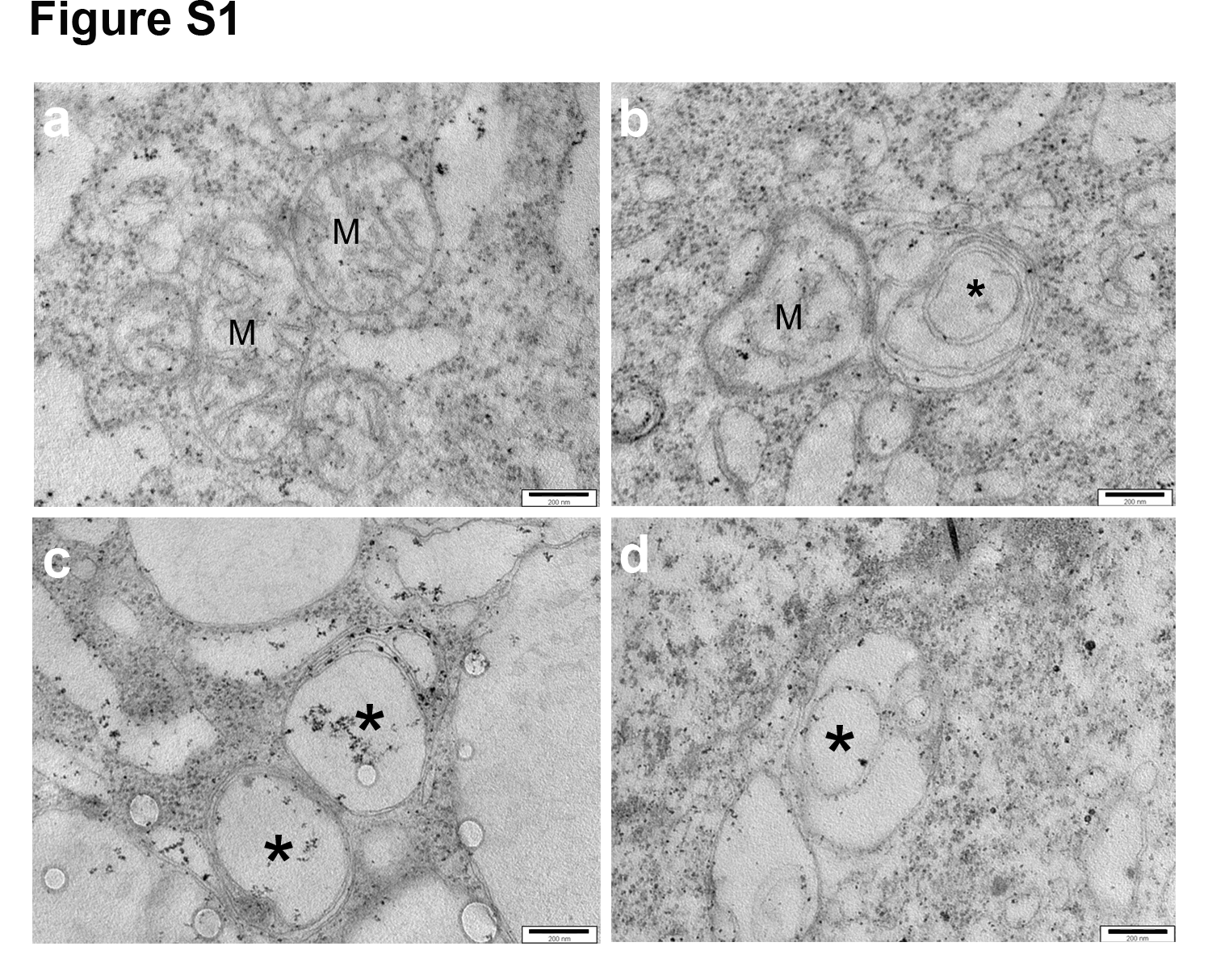

Supplement: Figure S1 — Abnormal mitochondrial structure in spc/spc spinal cord motor neurons. Representative electron micrographs of mitochondria (M) in motor neuron cell bodies from WT (a) and spc/spc mice (b-d). Asterisks indicate abnormally stacked or concentric membranes. Scalebar: 200 nm. (TIF) [file pone.0104237.s001.tif]

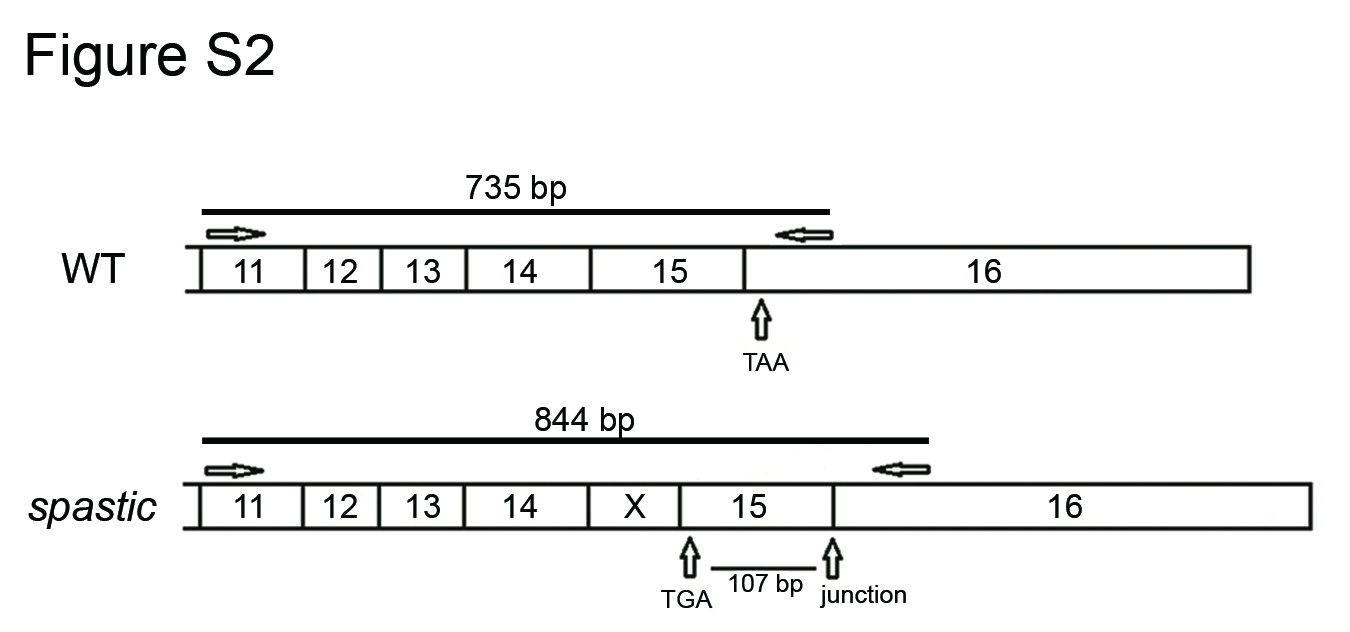

Supplement: Figure S2 — Exon organization and primer pair positions of the sequenced DnaJC11 brain transcripts. Horizontal arrows denote the primer pair that was used for sequencing. Black horizontal lines denote the 735 and 844 base pairs PCR product from a wild type (WT) and a spastic mouse. The inserted 109 bp long additional exon in the spastic transcript is indicated with X. Stop codons of the two transcripts are indicated (TAA in WT, TGA in spastic). The 107 base pairs distance of the novel TGA stop codon in the spastic transcript from its next downstream exon-exon junction is also indicated. (TIF) [file pone.0104237.s002.tif]

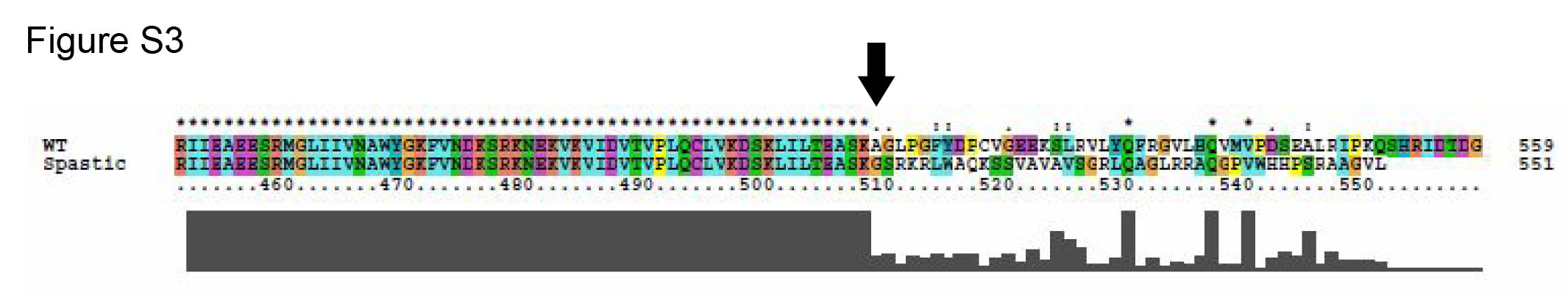

Supplement: Figure S3 — Protein sequence alignment of the C-terminal region of the WT and predicted mutant (Spastic) DNAJC11. Grey bars denote percentage of conservancy. Asterisks denote 100% conservancy. Black arrow denotes the position of the first frameshifted amino acid of the predicted mutant protein. (TIF) [file pone.0104237.s003.tif]

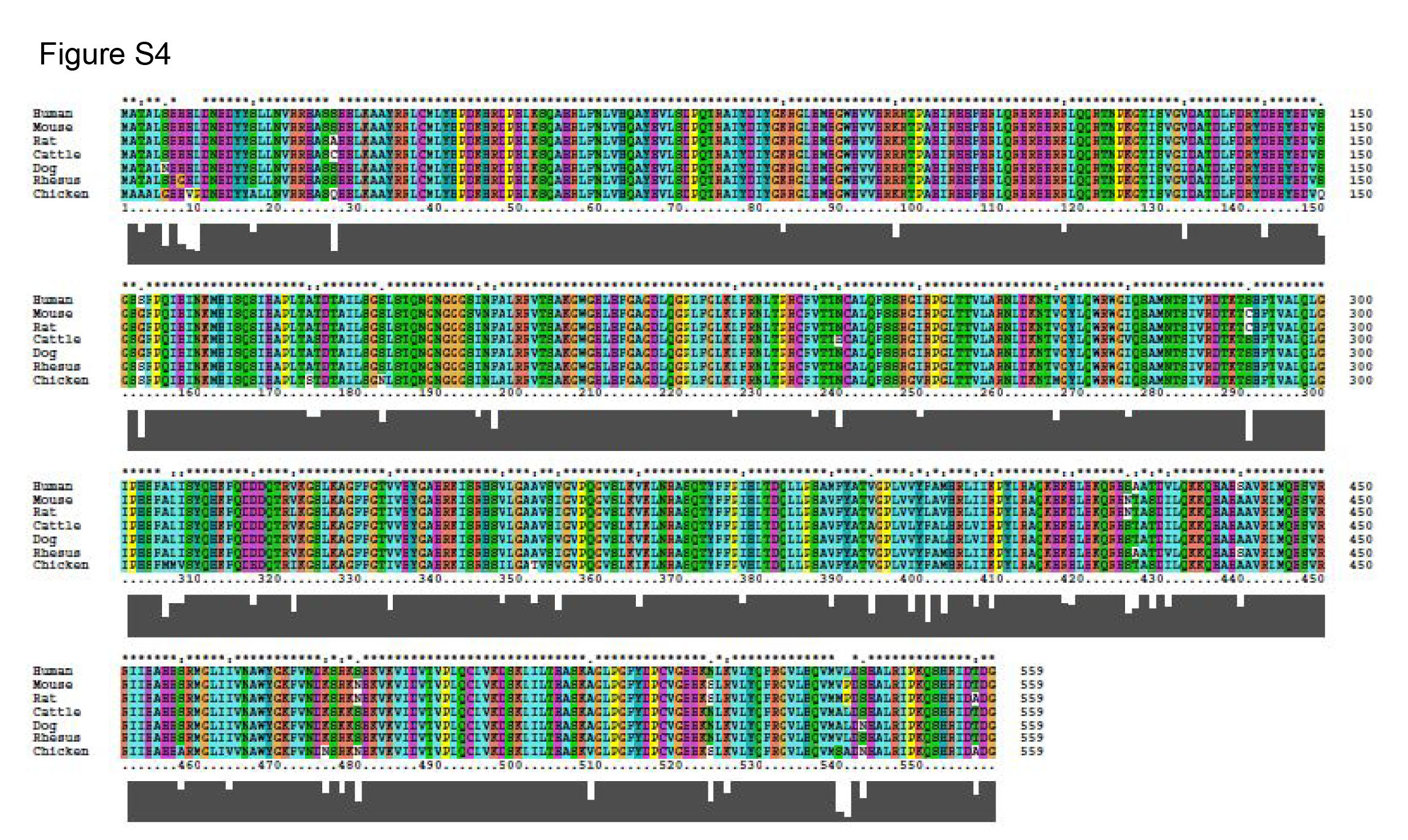

Supplement: Figure S4 — Multiple sequence alignment of the DNAJC11 63 kDa isoforms between the indicated vertebrate species. Sequences were obtained from Ensembl database. Grey bars denote percentage of conservancy. Asterisks denote 100% conservancy. (TIF) [file pone.0104237.s004.tif]

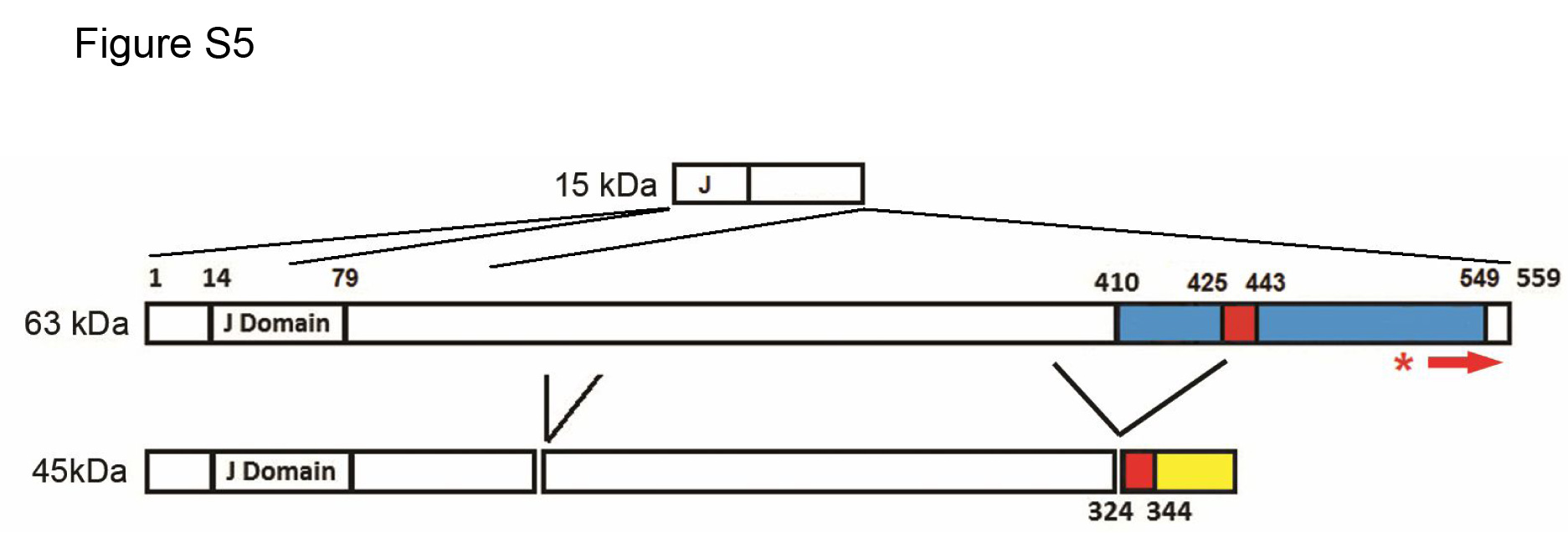

Supplement: Figure S5 — Schematic diagram of the three predicted isoforms of muDNAJC11. Amino acid numbers for all recognized domains are shown. Region in blue represents the DUF3395 domain. Region in red represents the coiled coil domain. Red asterisk and red arrow represent the site of the mutation and the stretch of the predicted mutated sequence respectively. Yellow region in the 45 kDa isoform represents a region absent in the other isoforms. Black lines denote the protein regions of the 63 kDa isoform which are absent in the other isoforms. (TIF) [file pone.0104237.s005.tif]

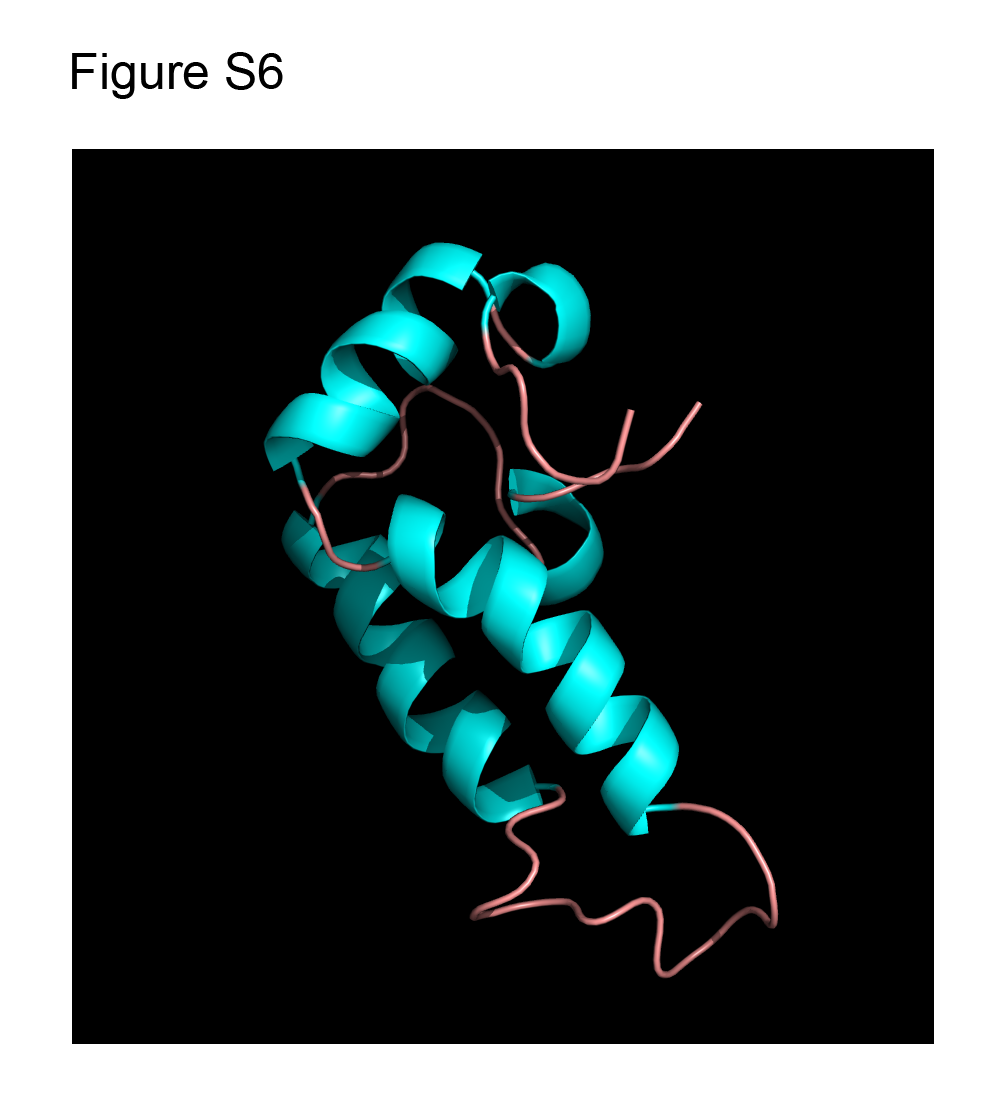

Supplement: Figure S6 — A structural model of the J domain of the N-terminal 14–72 amino acid residues of DNAJC11. A-helices are represented as cyan springs and non ordered linkers as purple tubes. (TIF) [file pone.0104237.s006.tif]

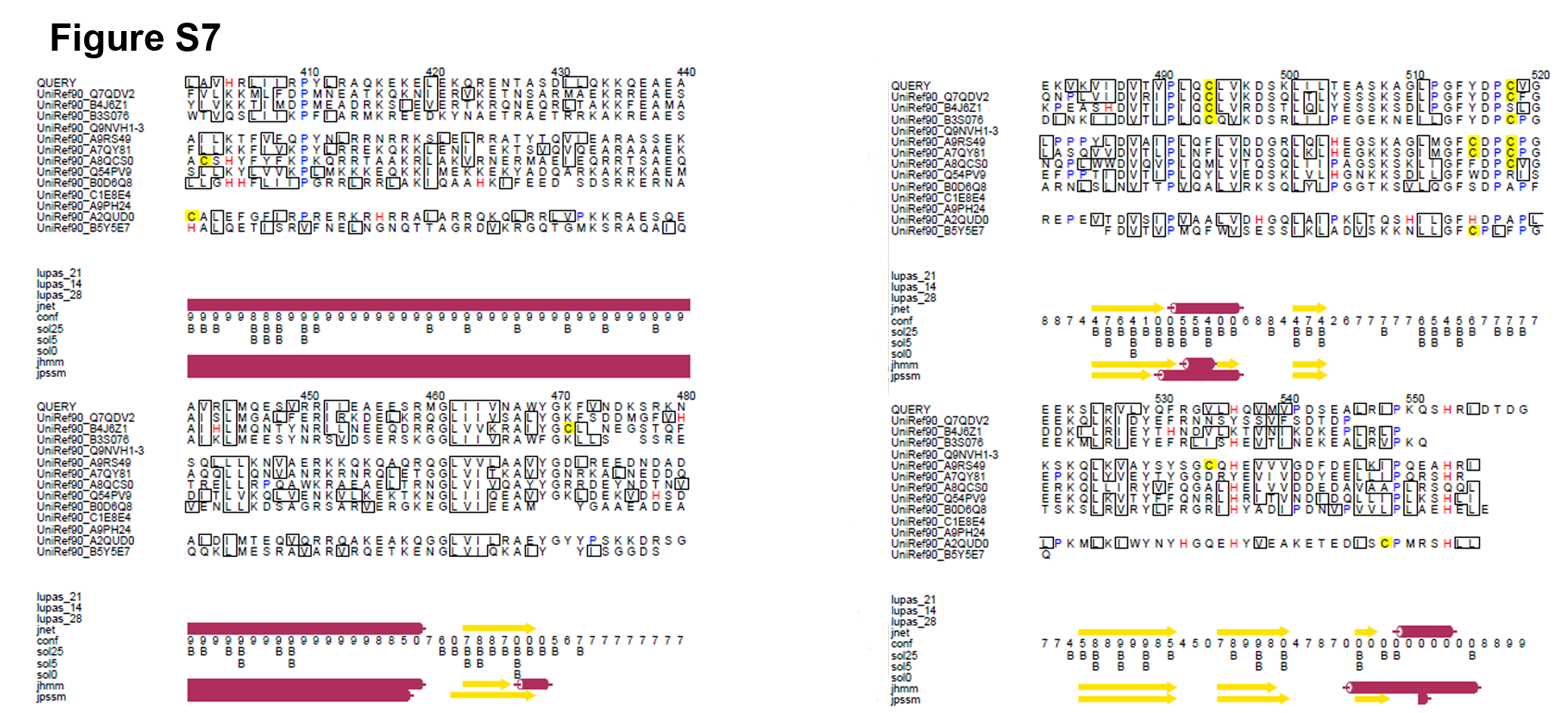

Supplement: Figure S7 — The secondary structure prediction for the C-terminal region of the 63 kDa isoform. Each block runs horizontally along the protein's amino acid sequence and vertically indicating homologues sequence analysis, sequence conservation (boxes), and predicted secondary structure elements (α- helices as purple rods and β-strands as yellow arrows). Query, Mus musculus; Q7QDV2, Anopheles gambiae; B4J6Z1, Drosophila grimshawi; B3S076, Trichoplax adhaerens; Q9NVH, Homo sapiens; A9RS49, Physcomitrella patens; A8QCS0, Malassezia globosa; Q54PV9, Dictyostelium discoideum; B0D6Q8, Laccaria bicolor; C1E8E4, Micromonas sp.; A9PH24, Populus trichocarpa; A2QUD0, Aspergillus niger. (TIF) [file pone.0104237.s007.tif]

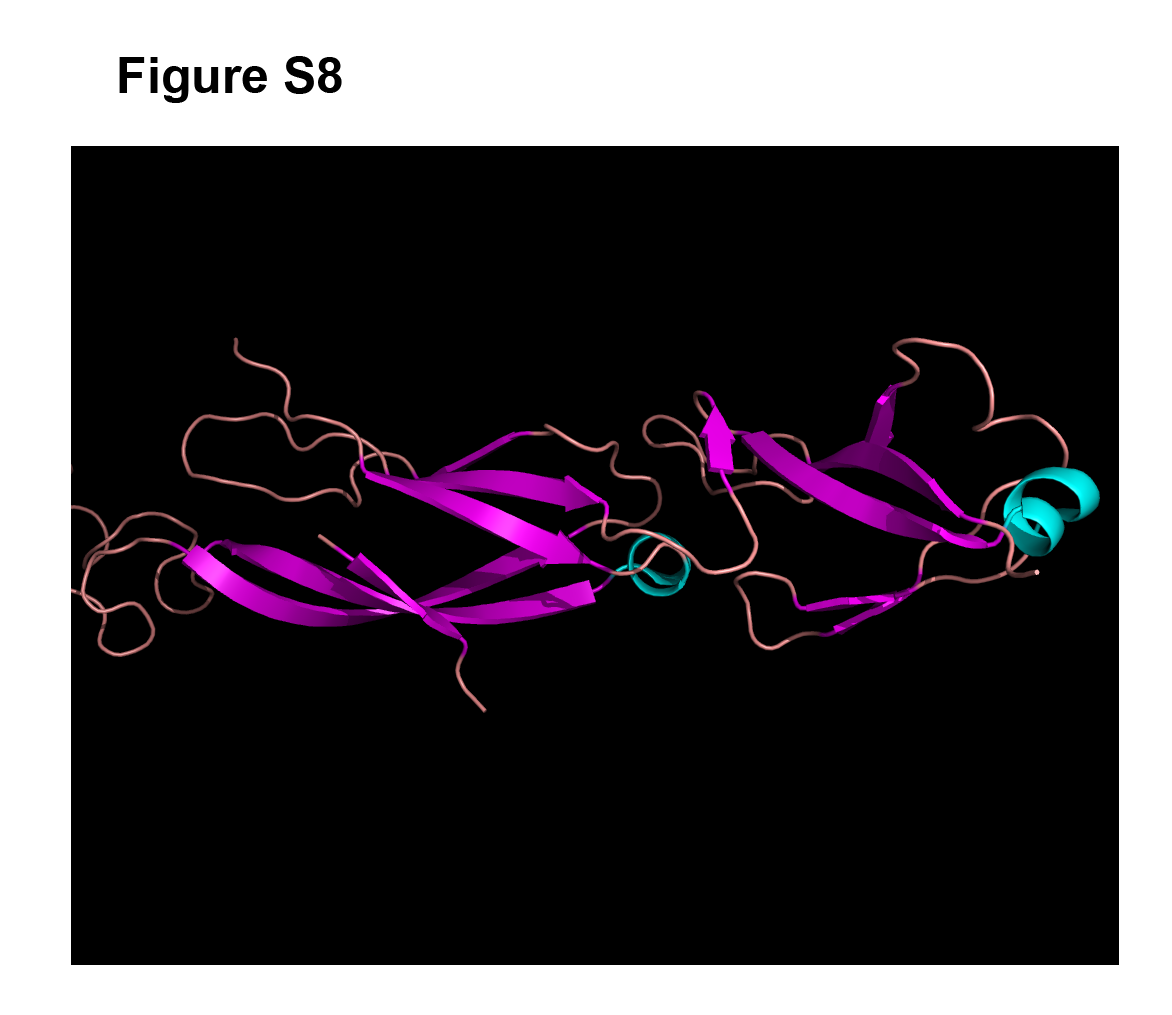

Supplement: Figure S8 — A structural model of the C-terminal domain of the protein MAS5/HSP40/YDJ1. This model resembles the C-terminal domain of the 63 kDa isoform of muDNAJC11 that may form the association domain. Α-helices are shown in cyan, β-strands are shown in purple. On the left, part of the coiled coil region is shown, represented as pink tube. (TIF) [file pone.0104237.s008.tif]

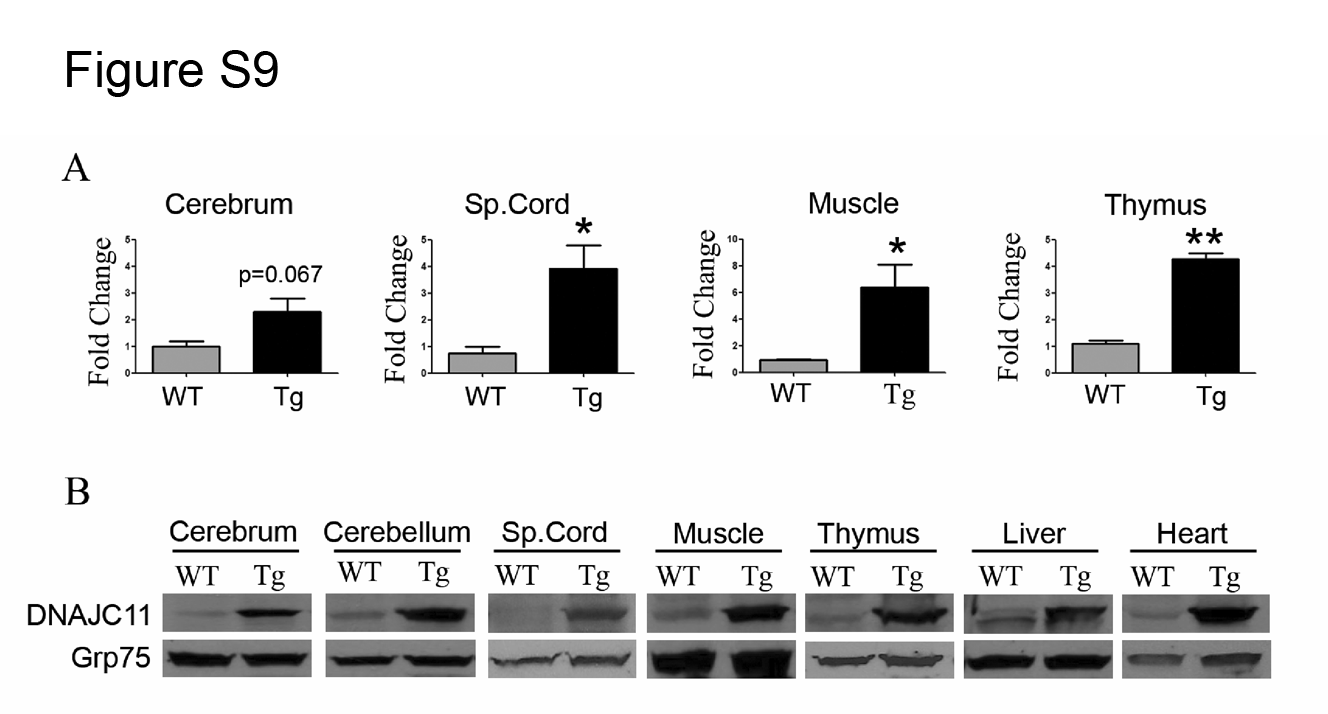

Supplement: Figure S9 — Expression analysis of DnaJC11 in TgF869 transgenic mice. (A) qPCR analysis of DnaJC11 expression using a primer pair common for both mouse and human DnaJC11 transcripts. Cerebrum, Wt (n = 5), Tg (n = 8); Spinal Cord, WT (n = 3), Tg (n = 6); Muscle (n = 4); Thymus (n = 2-3). Data represent mean ± SE. (B) Western blot analysis of isolated mitochondria for DNAJC11 expression in various tissues of WT and Tg mice. Grp75 served as a loading control. Student's t test was performed for statistical analysis. ***p< 0.001, **p<0.01, *p<0.05. (TIF) [file pone.0104237.s009.tif]

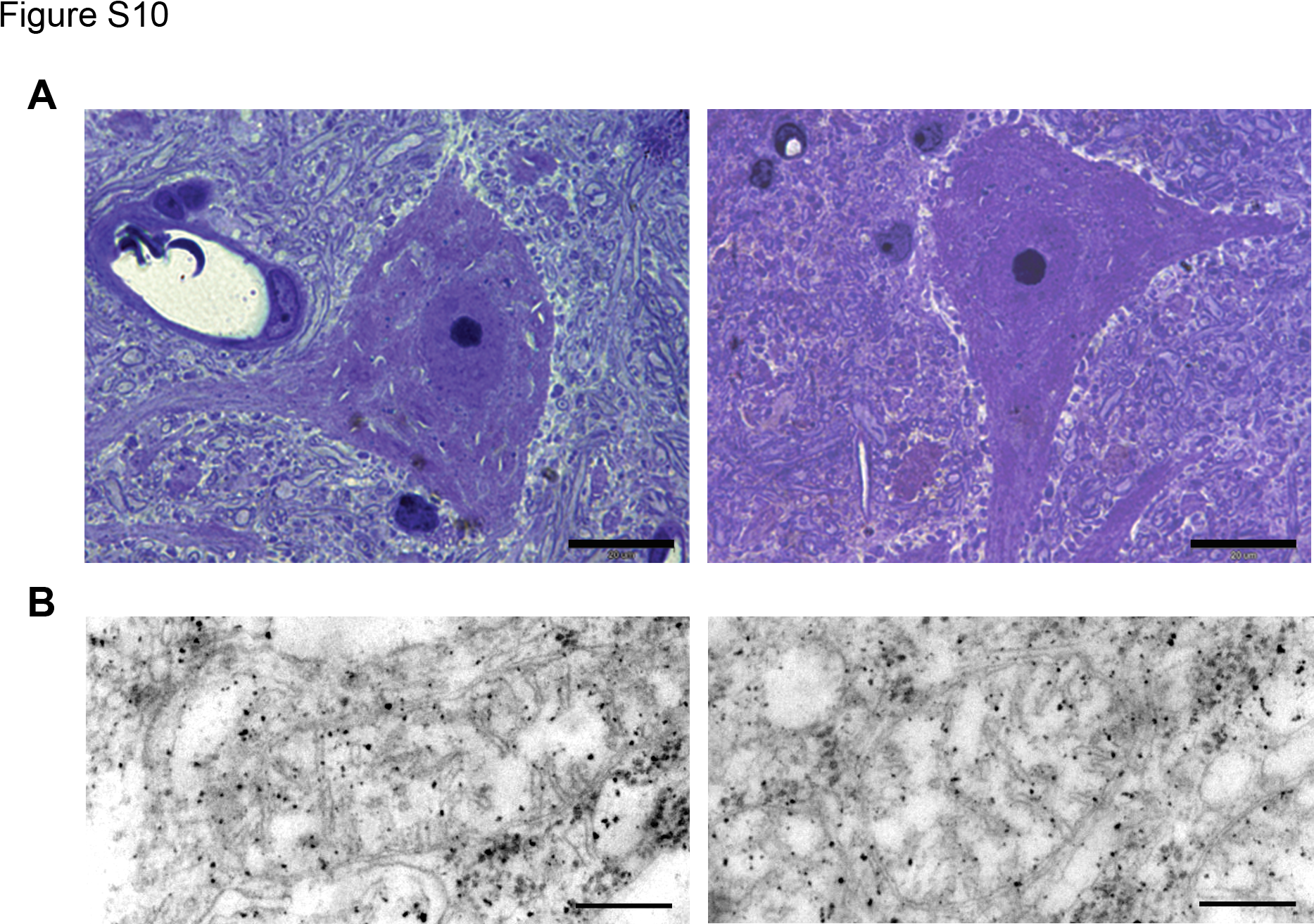

Supplement: Figure S10 — Normal motor neuron morphology in rescued (TgF869/ DnaJC11spc/spc ) mice. (A) Representative toluidine blue stained semi-thin resin sections showing motor neurons in the ventral horn of the spinal cord from two, 2 month old rescued mice. Motor neurons of rescued mice (n = 2) had a perfectly normal appearance and were indistinguishable from the WT ones. Scalebar: 20 µm. (B) Representative electron micrographs of mitochondria in motor neuron cell bodies from rescued mice. Scalebar: 200 nm. (TIF) [file pone.0104237.s010.tif]
