# Supplementary material for: A Splicing Mutation in the Novel Mitochondrial Protein DNAJC11 Causes Motor Neuron Pathology Associated with Cristae Disorganization, and Lymphoid Abnormalities in Mice
Source: PLoS One. 2014 Aug 11;9(8):e104237. doi: 10.1371/journal.pone.0104237 (PMC4128653; doi:10.1371/journal.pone.0104237)
Supplement: Table S1 — Fine mapping results. (DOC) [file pone.0104237.s011.doc]

**Table S1:** Fine mapping results.

| **Marker** | **Recombinations** | | | | | | | | | **Mbp** |
| --- | --- | --- | --- | --- | --- | --- | --- | --- | --- | --- |
| D4Mit189 | B | B | B | B | B | H | H | H | H | 147,95 |
| D4Mit33 | B | B | B | B | B | H | H | H | H | 149,96 |
| **rs3665061** | B | B | B | B | B | B | B | B | B | **150,54** |
| D4Mit42 | B | B | B | H | H | B | B | B | B | 150,94 |
| **rs32610416** | H | H | H | H | H |  | B | B | B | **152,54** |
|  | *1514* | *2608* | *113* | 1604 | 2426 | *1405* | *1406* | *1408* | *2093* |  |

Polymorphic markers and chromosome position on distal chromosome 4 are shown in the left and right columns respectively. The two candidate region defining markers and respective chromosome positions are in bold. Bottom row are individual mice code numbers (italics and regular fonts indicate *spastic* and healthy respectively). In Recombinations boxes, B denotes homozygote for the mutagenesis strain and H denotes heterozygous or homozygous for the mapping strain. Two healthy control mice (1604 and 2426) were found homozygous for SNP rs3665061 and three *spastic* mice (1514, 2608 and 113) were found heterozygous for SNP rs32610416, indicating that the causal mutation resides within this region.
